# EBV/HHV-6A dUTPases contribute to myalgic encephalomyelitis/chronic fatigue syndrome pathophysiology by enhancing T_FH_ cell differentiation and extrafollicular activities

**DOI:** 10.1172/jci.insight.158193

**Published:** 2022-06-08

**Authors:** Brandon S. Cox, Khaled Alharshawi, Irene Mena-Palomo, William P. Lafuse, Maria Eugenia Ariza

**Affiliations:** 1 Department of Cancer Biology and Genetics,; 2 Department of Microbial Infection and Immunity, and; 3 Institute for Behavioral Medicine Research, The Ohio State University Wexner Medical Center, Columbus, Ohio, USA.

**Keywords:** Infectious disease, Cellular immune response, Cytokines, NKT cells

## Abstract

Myalgic encephalomyelitis/chronic fatigue syndrome (ME/CFS) is a chronic, debilitating, multisystem illness of unknown etiology for which no cure and no diagnostic tests are available. Despite increasing evidence implicating EBV and human herpesvirus 6A (HHV-6A) as potential causative infectious agents in a subset of patients with ME/CFS, few mechanistic studies address a causal relationship. In this study we examined a large ME/CFS cohort and controls and demonstrated a significant increase in activin A and IL-21 serum levels, which correlated with seropositivity for antibodies against the EBV and HHV-6 protein deoxyuridine triphosphate nucleotidohydrolase (dUTPases) but no increase in CXCL13. These cytokines are critical for T follicular helper (T_FH_) cell differentiation and for the generation of high-affinity antibodies and long-lived plasma cells. Notably, ME/CFS serum was sufficient to drive T_FH_ cell differentiation via an activin A–dependent mechanism. The lack of simultaneous CXCL13 increase with IL-21 indicates impaired T_FH_ function in ME/CFS. In vitro studies revealed that virus dUTPases strongly induced activin A secretion while in vivo, EBV dUTPase induced the formation of splenic marginal zone B and invariant NKT_FH_ cells. Together, our data indicate abnormal germinal center (GC) activity in participants with ME/CFS and highlight a mechanism by which EBV and HHV6 dUTPases may alter GC and extrafollicular antibody responses.

## Graphical abstract

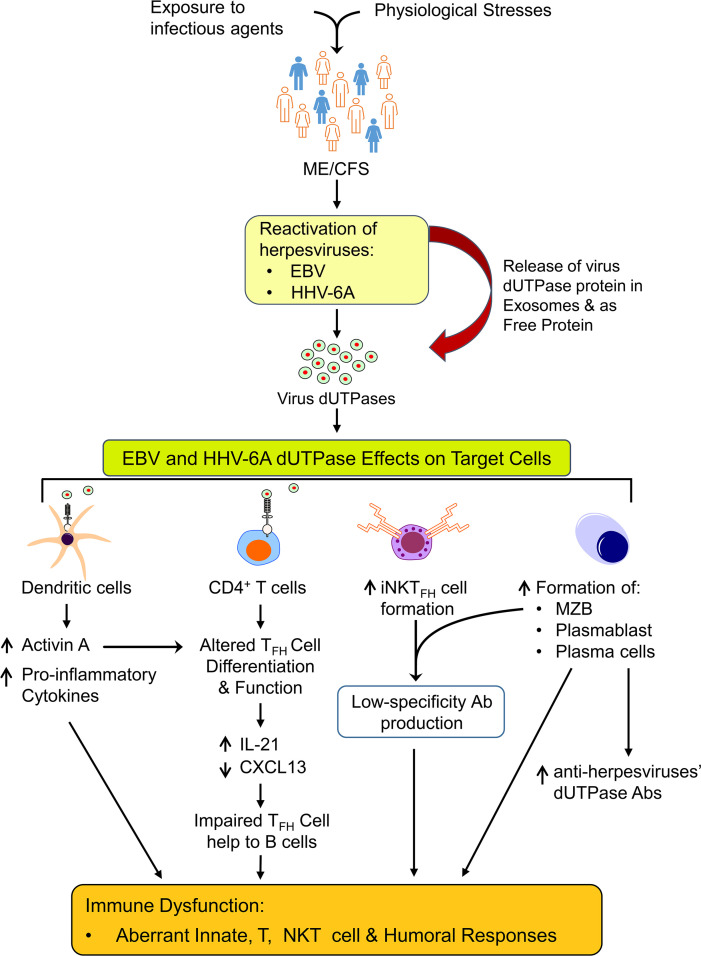

## Introduction

Myalgic encephalomyelitis/chronic fatigue syndrome (ME/CFS) is a chronic multisystem illness of unconfirmed etiology. ME/CFS has largely been diagnosed based upon the Fukuda CFS criteria (1) and/or Canadian Consensus ME/CFS criteria (2). In 2015 the National Academy of Medicine (formerly the Institute of Medicine) proposed a new name, systemic exertion intolerance disease, and case criteria focused on exhibiting chronic fatigue, post-exertional malaise, and orthostatic intolerance or cognitive deficits (3). No biomarker associations have been established in ME/CFS, and a diagnosis relies solely on symptom-based exclusion criteria.

Numerous mechanisms have been hypothesized to explain the pathology of ME/CFS, including autoimmunity, chronic infection, energy metabolic defect, and endocrine and neurological disturbances, but at this time the etiology of ME/CFS is unknown. An increase in autoantibodies against cellular neurotransmitters, neurotransmitter receptors, and the human deoxyuridine triphosphate nucleotidohydrolase (dUTPase) proteins (4–8) possibly triggered following an infection (9) has recently been reported. Several viruses, including EBV and human herpesvirus 6A (HHV-6A), have been implicated in ME/CFS pathophysiology (10). However, establishing a causal relationship between a virus and ME/CFS has been challenging because of the heterogeneity of the patient population, the high prevalence of these viruses in the population, and the existence of possibly multiple etiologies for ME/CFS.

EBV and HHV-6A/6B infect a significant percentage (>95%) of the worldwide population and establish lifelong persistent infections. Epidemiological, serological, and viral titer studies have implicated EBV and HHV-6A/B in the pathogenesis of several autoimmune diseases, including rheumatoid arthritis, Sjögren’s syndrome, systemic lupus erythematosus, and multiple sclerosis. EBV has also been linked to the development of several human B cell malignancies (AIDS/post-transplantation lymphoma, Burkitt’s lymphoma, Hodgkin’s/non-Hodgkin’s lymphoma) and epithelial carcinomas (nasopharyngeal carcinoma, gastric carcinomas) (8, 11–16).

We have previously shown that the EBV and HHV-6A dUTPases, produced by these viruses during abortive lytic and lytic replication, function as pathogen-associated molecular pattern proteins for TLR2. Engagement of TLR2 by both dUTPases leads to NF-κB activation and subsequent modulation of downstream genes involved in innate and adaptive immune responses (8, 11–16). Further studies using human clinical specimens demonstrated that EBV dUTPase might exacerbate the immunopathology in some lupus nephritis cases (15) and a subgroup of individuals with ME/CFS and Gulf War illness (GWI) (8), 2 multisystem diseases with overlapping symptomology. Notably, approximately 50% of participants with ME/CFS exhibit increased antibody levels for the dUTPases of multiple herpesviruses, including EBV and HHV-6A (8). These results support a role for virus dUTPase proteins in the pathologies associated with EBV and HHV-6A infections.

In this study we continued to investigate the mechanism(s) by which EBV and HHV-6A dUTPase proteins contribute to the immune dysfunction observed in ME/CFS and identified activin A, an early regulator of human follicular helper T cell (T_FH_) differentiation, as well as IL-21, a regulator of germinal center (GC) T_FH_ cell survival and plasma cell differentiation, to be markedly increased in ME/CFS sera. The lack of simultaneous increase of CXCL13 with IL-21 indicates impaired T_FH_ function in ME/CFS. While an increase in T_FH_ and circulating T_FH_ cells has been reported in individuals with infectious mononucleosis, a disease caused by EBV (17, 18), there have not been any studies to determine whether T_FH_ cells are altered in ME/CFS or following HHV-6A infection.

Our data highlight a potentially novel, unexplored mechanism in ME/CFS disease consistent with abnormal GC and extrafollicular antibody responses induced by a herpesvirus protein, which may contribute to autoantibody development in a subset of individuals with ME/CFS.

## Results

### ME/CFS sera exhibit heightened levels of activin A and IL-21.

**Figure 1:**
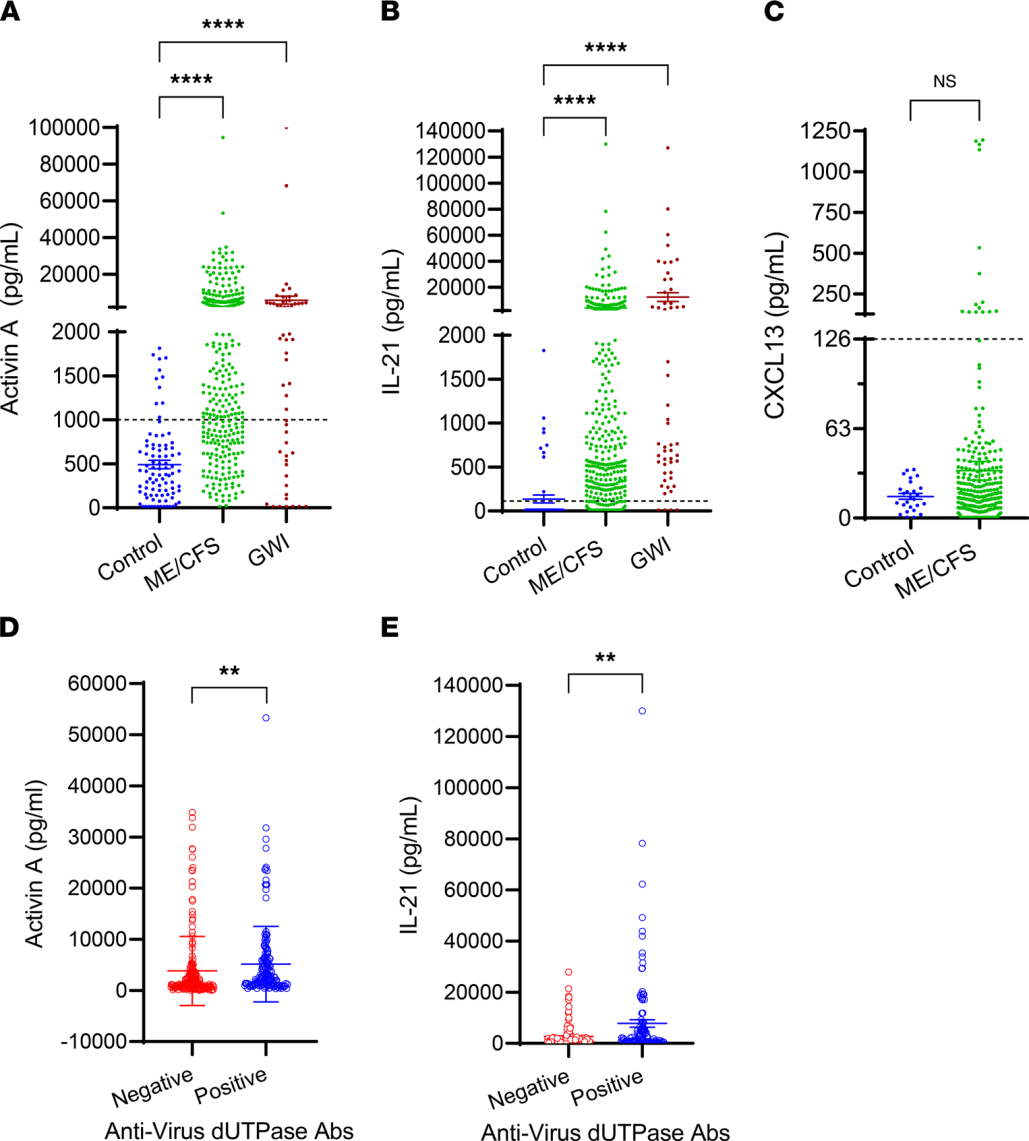
Patients with ME/CFS exhibit heightened serum levels of activin A and IL-21, which positively correlate with increased anti-herpesvirus dUTPase Abs. ELISA of (**A**) activin A and (**B**) IL-21 in serum of ME/CFS cases (*n* = 351), GWI veterans (*n* = 54), and healthy controls (*n* = 77). (**C**) Serum CXCL13 ELISA of ME/CFS cases (*n* = 351) and healthy controls (*n* = 27). (**D**) Comparison of activin A levels between ME/CFS cases positive for anti-herpesvirus dUTPase Abs (*n* = 167) versus negative (*n* = 184). (**E**) Comparison of IL-21 levels between ME/CFS cases (*n* = 347) positive (*n* = 157) versus negative (*n* = 190) for Abs against the dUTPases from herpesviruses. Dotted line represents the normal range levels for healthy individuals for each cytokine/chemokine. Data represent 3 experiments with mean ± SEM. (**A** and **B**) ****P < 0.0001 of disease versus control cohorts by 1-way ANOVA Kruskal-Wallis multiple comparisons test, **P < 0.01 of anti-virus dUTPase Ab–positive versus –negative groups (**D** and **E**) by 2-tailed Mann-Whitney *U* test.

Microarray gene expression analysis of EBV dUTPase in human dendritic cells (hDCs) revealed a strong induction of inhibin subunit beta A (13), which encodes activin A. This finding along with the presence of heightened Ab levels against the dUTPase of multiple herpesviruses (8) suggest that EBV dUTPase might contribute to the immune dysfunction observed in ME/CFS through activin A, a potent inducer of T_FH_ cell differentiation in humans (19). To address this possibility, we measured serum activin A levels in 351 individuals with ME/CFS, 54 veterans with GWI, and 77 control individuals (Table 1). Of all ME/CFS cases examined, 67% (*n* = 235) had levels of activin A above the normal range (0–1000 pg/mL) compared with only 14% (*n* = 11) of controls. As shown in Figure 1A, a significant increase in the concentration of activin A was observed in ME/CFS cases, ranging from 1014.03 to 94,613 pg/mL, compared with the controls (1029–1813 pg/mL, *P* < 0.0001). Kruskal-Wallis multiple-comparison analysis of activin A serum levels across all 3 cohorts revealed that participants with GWI exhibited significantly increased levels of activin A, ranging from 1121 to 100,000 pg/mL (63%, *n* = 34, *P* < 0.0001), relative to the controls and comparable to those exhibited in ME/CFS (Figure 1A). Since GC T_FH_ cells are functionally mature helpers to B cells and are characterized by high expression of IL-21 and CXCL13, we next sought to examine the ME/CFS sera for the presence of IL-21 and CXCL13. A similarly increased pattern to that of activin A was observed for IL-21–positive cases relative to the controls (Figure 1B). Within the ME/CFS cohort, 67 individuals expressed IL-21 concentrations within normal levels (0–115 pg/mL), and 302 exhibited significantly heightened IL-21 levels, ranging from 125.52 to 130,000 pg/mL (86%, *P* < 0.0001), while in the control cohort, only 9 individuals (11.68%) had IL-21 levels above the normal range. Examination of the GWI veterans’ cohort showed a significant increase in serum IL-21 levels, ranging from 197.54 to 127,000 pg/mL (92.45%, *n* = 49), compared with the control cohort (*P* < 0.0001) (Figure 1B). When we examined the ME/CFS cohort for the presence of CXCL13 (Figure 1C), only 14 out of 351 individuals exhibited CXCL13 levels above normal (>126 pg/mL, ranging from 139.42 to 1195.1 pg/mL). The remaining participants with ME/CFS expressed CXCL13 levels within the normal range (0.43 to 126 pg/mL). Comparison of CXCL13 serum levels between the ME/CFS and control cohorts revealed no significant difference (mean ± SEM 33.29 ± 126.7 versus 15.27 ± 10.41, *P* = 0.2152 by 2-tailed Mann-Whitney *U* test). Furthermore, of the ME/CFS serum samples examined, 23 exhibited high levels (>4000 pg/mL) of both activin A and IL-21. We next evaluated whether a correlation could be established between seropositivity for EBV and/or HHV-6A dUTPase Abs and serum levels of activin A and/or IL-21 within the ME/CFS cohort. Of the samples examined for activin A or IL-21, 47.18% (*n* = 167) and 45.24% (*n* = 157) respectively, were positive for Abs against HHV-6A, EBV, or both dUTPases. As shown in Figure 1D, the levels of serum activin A were significantly higher in ME/CFS cases seropositive for Abs against the virus dUTPases (5156 pg/mL ± 585.1) than in seronegative cases (3809 pg/mL ± 500.8, *P* = 0.0353). Likewise, a significant difference was observed in IL-21 levels in ME/CFS cases seropositive for virus dUTPases’ Abs compared with virus-seronegative cases (7818 ± 1481 versus 2672 ± 356.5, *P* = 0.0014) (Figure 1E).

### ME/CFS sera induces T_FH_ differentiation of naive CD4^+^ T cells.

**Figure 2:**
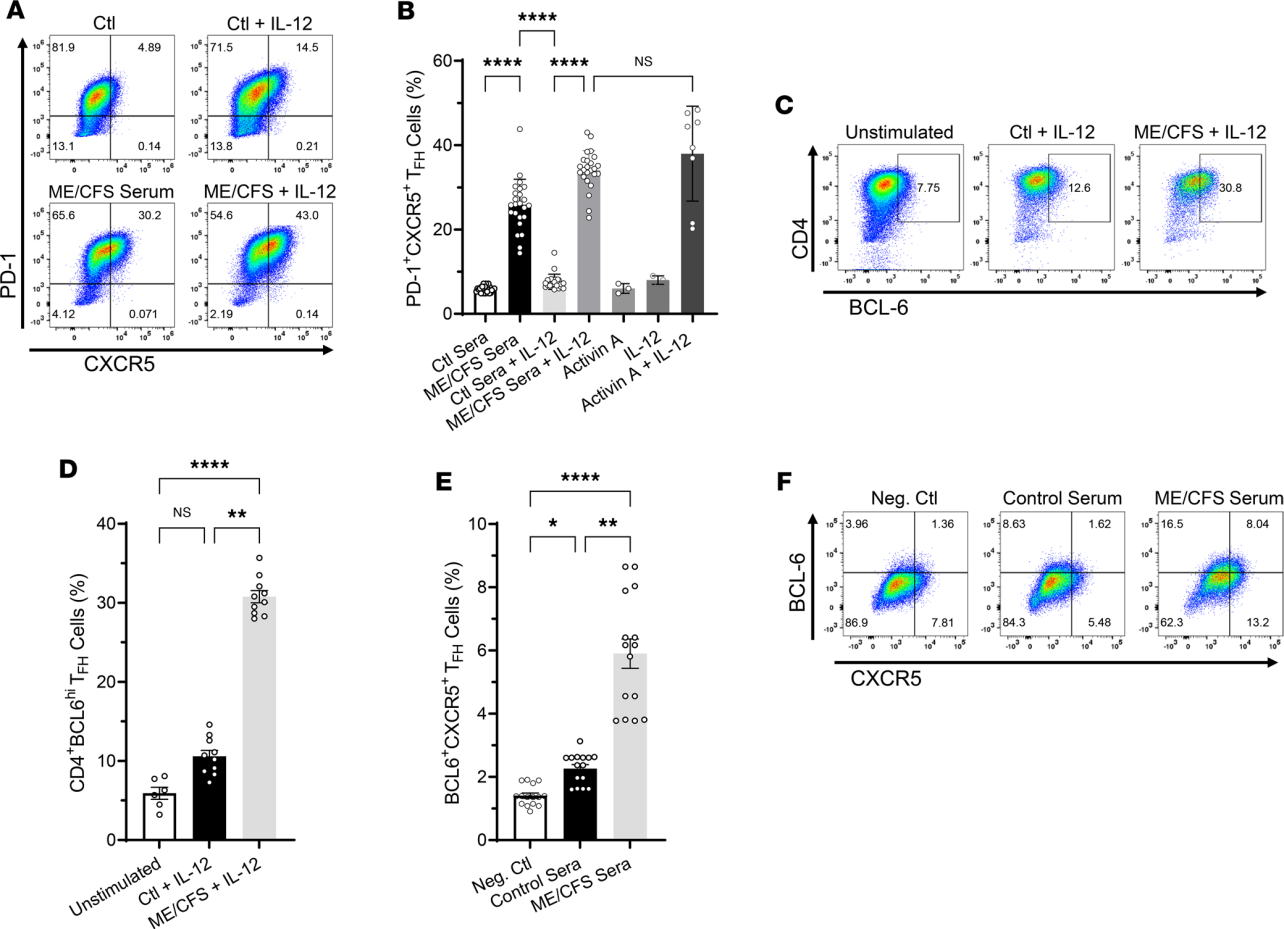
ME/CFS sera drive T_FH_ cell differentiation of human naive CD4^+^ T cells in vitro. Naive CD4^+^ T cells (CD4^+^CD45RA^+^CD197^+^) were isolated from human PBMCs by magnetic bead negative selection and cultured in vitro with anti-CD3/anti-CD28–coated beads in the presence of control or ME/CFS sera (2.5% vol/vol) with or without IL-12 (5 ng/mL), or activin A (Act A; 100 ng/mL) with or without IL-12 and IL-12 only. After 3 days, cells were examined for expression of the T_FH_ markers PD-1, CXCR5, and BCL6 by FACS analysis. (**A**) Representative FACS plots indicating PD-1^+^CXCR5^+^ T_FH_ cell frequency (upper right quadrant) and (**B**) quantitative bar graph of percentage of PD-1^+^CXCR5^+^ cells following human naive CD4^+^ T cell stimulation for 3 days as described above; *n* = 24 activin A and IL-12, controls *n* = 3–8. Cells activated with anti-CD3/anti-CD28 beads in the presence of activin A plus IL-12 were used as positive control for T_FH_ cell differentiation, while cells stimulated with beads only were used as the negative control. (**C**–**F**). Frequency of CD4^+^BCL6^+^ T_FH_ cells (**C**) and BCL6^+^CXCR5^+^ T_FH_ cells (**F**; upper right quadrants). Quantitative bar graph of percentage of CD4^+^BCL6^+^ *n* = 6–10 (**D**) and BCL6^+^CXCR5^+^ *n* = 15 (**E**) cells differentiated as described above. FACS samples were pregated on CD4^+^ and fixable viability dye eFluor 780 negative (live cells). Data represent 3 independent experiments with mean ± SEM. *P < 0.05, **P < 0.01, ****P < 0.0001 of treatment groups versus controls by 1-way ANOVA Kruskal-Wallis multiple comparisons test with Dunn’s correction.

**Figure 3:**
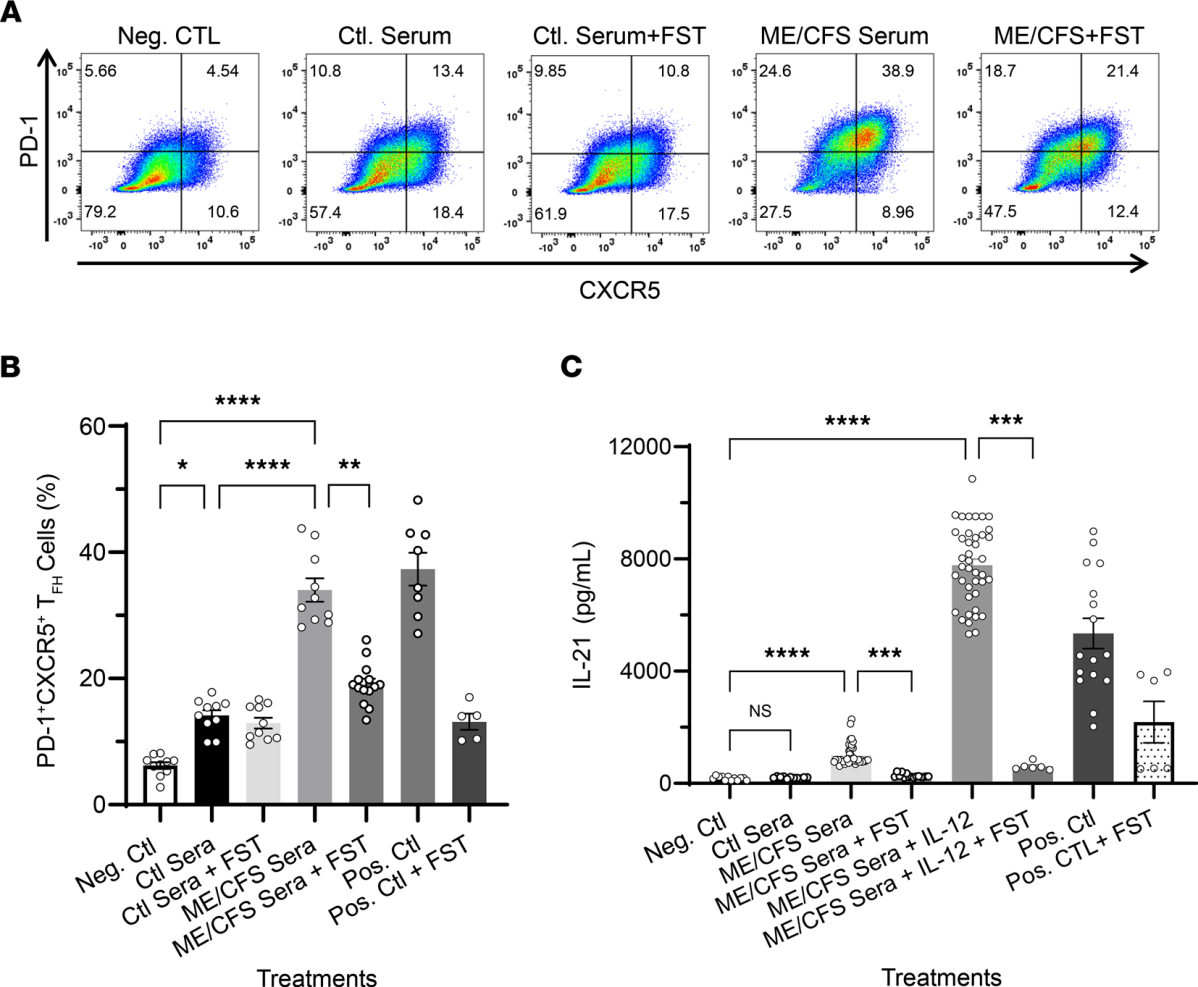
ME/CFS sera–induced T_FH_ cell differentiation of human naive CD4^+^ T cells is mediated by activin A. For human activin blocking experiments, dUTPase-derived or control DC-conditioned media (25% vol/vol) as well as ME/CFS or control sera (2.5% vol/vol) were preincubated with follistatin-315 (FST, 1 μg/mL) or vehicle (DMSO) for 1 hours, added to freshly isolated naive CD4^+^ T cells, and incubated for 3 days at 37°C. On day 4, supernatants and cells were collected for analysis of IL-21 levels in the presence or absence of FST by ELISA and T_FH_ differentiation blockade by FACS, respectively. Activin A + IL-12 treatment in the presence of FST was used as a positive control for T_FH_ differentiation blockade, and bead-treated cells were used as a negative control. Activin A + IL-12 treatment was used as a positive control for inducing T_FH_ cell differentiation. (**A**) Representative flow cytometry plots indicating the frequency of PD-1^+^CXCR5^+^ double-positive T_FH_ cells (upper right quadrants) and (**B**) quantitative bar graph of percentage of PD-1^+^CXCR5^+^ cells following human naive CD4^+^ T cell stimulation for 3 days as described above. (**C**) ELISA of IL-21 levels in supernatants of naive CD4^+^ T cells differentiated as described in **A** in the presence or absence of FST. Data represent a minimum of 3 experiments, with mean ± SEM. (**B** and **C**) *n* = 8–24, *P < 0.05, **P < 0.01, ***P < 0.001, ****P < 0.0001 of treatment groups versus negative control or control sera by 1-way ANOVA Kruskal-Wallis multiple comparisons test with Dunn’s correction.

To begin to elucidate the potential implications of heightened serum levels of potent regulators of human T_FH_ cell differentiation activin A and IL-21, we next tested whether serum from patients with ME/CFS could induce T_FH_ cell differentiation of naive CD4^+^ T cells in vitro. Flow cytometric analysis of human naive CD4^+^ T cells stimulated for 3 days with anti-CD3/CD28–coated beads alone or in the presence of ME/CFS patients’ sera (2.5% vol/vol), with or without IL-12, showed that all serum samples tested (*n* = 24) induced the expression of the T_FH_ cell signature markers programmed cell death 1 (PD-1) and CXCR5 (Figure 2A, bottom row representative plots, and Figure 2B) (up to 38.9% just by serum alone) compared with cells stimulated with anti-CD3/CD28–coated beads plus control serum (Figure 2A, representative top row plots, and Figure 2B). Interestingly, exogenous addition of IL-12 to ME/CFS sera further enhanced the frequency of cells exhibiting a T_FH_ cell–like phenotype overall. We also examined the frequency of cells with intranuclear expression of BCL6, the lineage-defining transcription factor of T_FH_ cells, and positive staining for the surface marker CXCR5 among cells differentiated with serum from ME/CFS and controls in the presence of IL-12 for 3 days. A significant fraction of CD4^+^ T cells differentiated with ME/CFS sera increased their expression of BCL6 (Figure 2, C and D) and CXCR5 (Figure 2, E and F) relative to that induced by the control sera. To determine whether the induction of T_FH_ signature markers by ME/CFS patient sera was activin A mediated, we conducted blocking experiments with follistatin-315 (FST), a natural inhibitor of activins. As shown in Figure 3, A and B, treatment of cells with FST plus serum from patients with ME/CFS for 3 days resulted in a significant decrease in the frequency of PD-1^+^CXCR5^+^ cells compared with cells treated with ME/CFS serum alone. Notably, this blockade in T_FH_ differentiation was accompanied by a significant decrease in IL-21 secretion in cells treated with FST compared with that induced by ME/CFS sera alone (Figure 3C). These results indicate that serum-mediated induction of T_FH_ differentiation is, in part, activin A dependent. These studies were conducted using serum-free medium conditions to eliminate the potential indirect effects of undefined serum components.

### EBV and HHV-6A dUTPase proteins strongly induce the secretion of regulators of T_FH_ cell differentiation.

**Figure 4:**
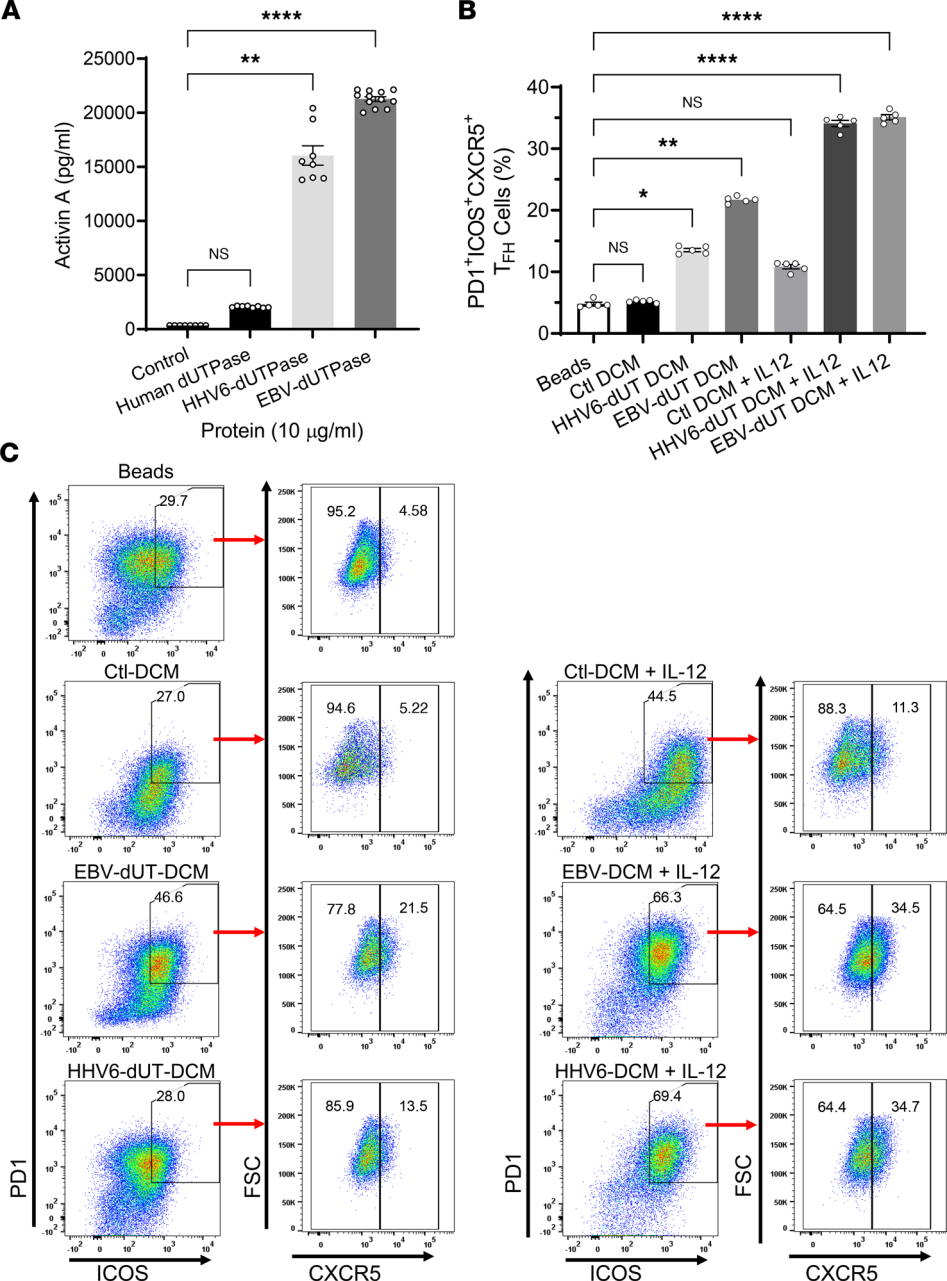
EBV and HHV-6A dUTPase–derived DCM are sufficient to induce in vitro T_FH_ cell differentiation of human naive CD4^+^ T cells. (**A**) Monocyte-derived hDCs (2.5 × 10^5^ cells) were stimulated with dUTPase protein (10 μg/mL) from HHV-6A or EBV or with the human nuclear dUTPase or left untreated (control) for 24 hours. After treatments, culture supernatants were examined for activin A concentration by ELISA. (**B** and **C**) Naive CD4^+^ T cells were isolated from PBMCs as described above (Figure 2 and Methods section) and cultured in vitro with anti-CD3/anti-CD28–coated beads alone (Beads) or in the presence of DCM (Ctl, HHV-6A dUTPase, or EBV dUTPase; 25% vol/vol), with or without IL-12. After 3 days, cells were examined for expression of the T_FH_ markers PD-1, ICOS, and CXCR5 by FACS. (**B**) Quantitative bar graph of percentage of PD-1^+^ICOS^+^CXCR5^+^ cells differentiated as described above. (**C**) Representative flow cytometry plots indicating the frequency of ICOS^+^PD-1^+^ double-positive cells expressing CXCR5 without IL-12 (left plots) or with IL-12 (far right plots). FACS analyses were conducted on the lymphocyte population, and samples were pregated on live (FVD^–^) CD4^+^ cells. Data represent the mean ± SEM of *n* = 8–12 (**A**) or *n* = 5 (**B** and **C**) independent experiments. *P < 0.05, **P = 0.01, ****P < 0.0001 of treatment groups versus control by 1-way ANOVA Kruskal-Wallis multiple comparisons test with Dunn’s correction.

We next evaluated the role of EBV/HHV-6A dUTPases as inducers of activin A, IL-21, and other regulatory factors that could contribute to the symptomology associated with ME/CFS. Analyses of primary hDC-conditioned media (DCM) following stimulation with EBV or HHV-6A dUTPase proteins for 24 hours showed that both herpesviruses’ dUTPase proteins strongly induced the secretion of activin A (21,279 ± 270.4 pg/mL, *P* = 0.005 for EBV; and 16,188 ± 1176 pg/mL, *P* = 0.0286 for HHV-6A) compared with the control (343.6 ± 3.586 pg/mL). The human control dUTPase induced a much weaker response (2062.2 ± 44.1 pg/mL) than EBV and HHV-6A dUTPases (10-fold and 7.85-fold less, respectively) (Figure 4A). Furthermore, the herpesviruses’ dUTPase proteins also induced the expression of B cell–activating factor (BAFF) (6.58 ± 0.69 and 12.40 ± 0.69 pg/mL for EBV and HHV-6A, respectively, versus 2.68 ± 0.8 pg/mL in control, *P* ≤ 0.02) and pentraxin-3 (3.44 ± 0.20 pg/mL and 2.93 ± 0.059 pg/mL for EBV and HHV-6A, respectively, versus 0.037 ± 0.005 pg/mL in control, *P* = 0.0001). These molecules are important for T_FH_ cell differentiation (19), for the proliferation and differentiation of B cells (20), and as adjuvants for marginal zone (MZ) B cells (21).

### EBV and HHV-6A dUTPase–derived DCM induce T_FH_ cell differentiation.

We next sought to determine whether DCM from EBV and HHV-6A dUTPase protein–treated hDCs could induce naive CD4^+^ T cells to differentiate into a T_FH_ cell–like phenotype. For this, naive CD4^+^ T cells were stimulated for 3 days with anti-CD3/anti-CD28–coated beads alone or in the presence of EBV dUTPase, HHV-6A dUTPase, or control DCM (25% vol/vol). After 3 days of culture, the expression of T_FH_ cell signature markers PD-1, ICOS, and CXCR5 was examined by flow cytometry analysis. As shown in Figure 4, B and C, EBV dUTPase DCM and HHV-6A dUTPase DCM alone were sufficient to induce the expression of PD-1, ICOS, and CXCR5 in a significant number of cells (Figure 4C, left plots) relative to that induced by control DCM or cells activated with anti-CD3/CD28–coated beads alone (Figure 4C, top plot). A potent induction of PD-1^+^ICOS^+^CXCR5^+^ cells was observed in response to EBV or HHV-6A dUTPase DCM together with IL-12 (Figure 4C, far right plots).

### EBV and HHV-6A dUTPase–derived DCM regulate T_FH_ cell function.

**Figure 5:**
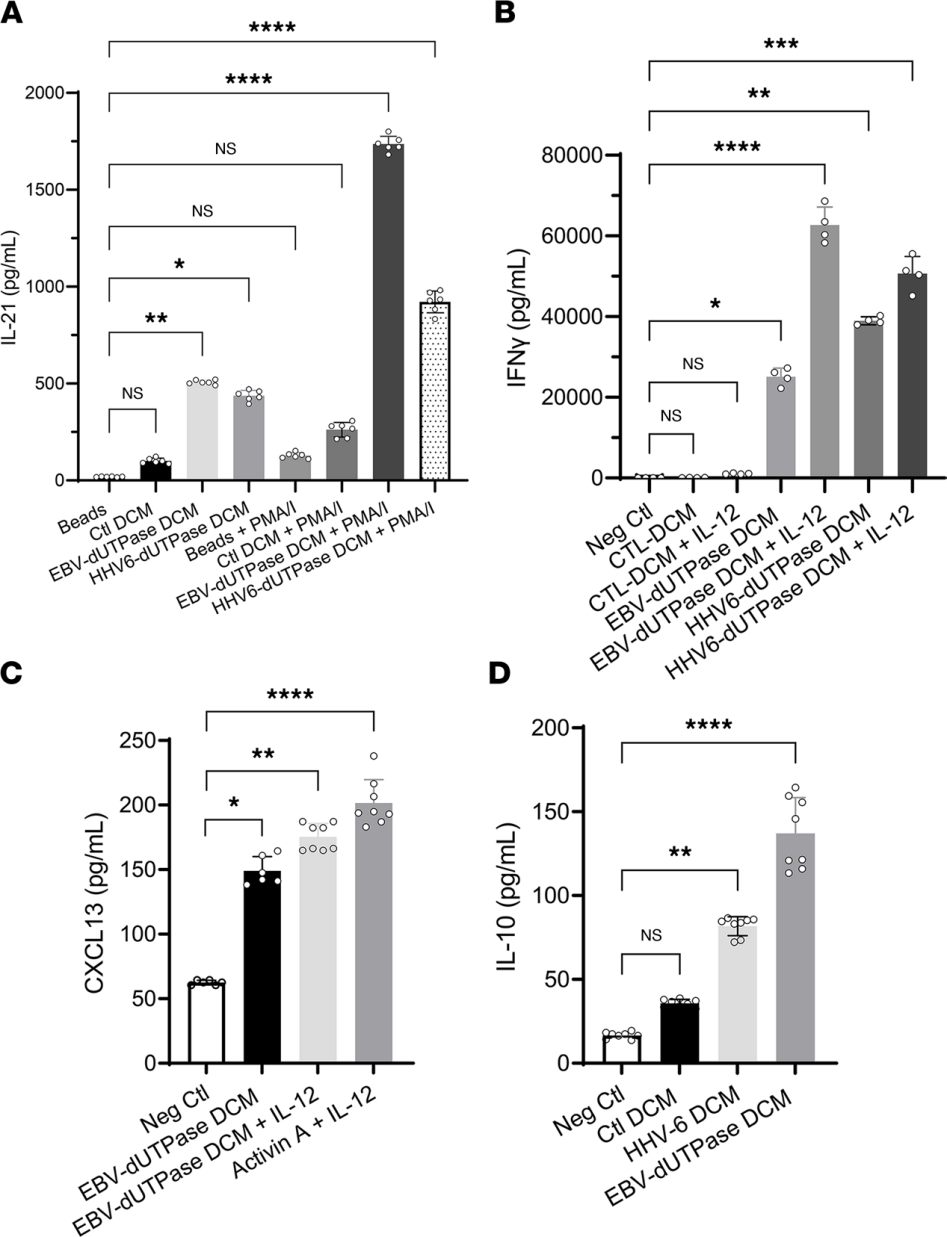
EBV and HHV-6A dUTPase DCM induce cytokines that are associated with T_FH_-like and T_H1_ cell signatures. (**A**) Naive CD4^+^ T cells were isolated from PBMCs as described above (Figure 2 and Methods section), cultured in vitro with anti-CD3/anti-CD28 coated beads alone (Beads) or in the presence of DCM (Ctl or EBV dUTPase or HHV-6A dUTPase; 25% vol/vol), and restimulated or not on day 3 with PMA/I. Following treatments, supernatants were examined for IL-21 (**A**) and IFN-γ (**B**) concentrations by ELISA. (**C** and **D**) ELISA of CXCL13 or IL-10 in supernatants of naive CD4^+^ T cells stimulated for 5 days by plate-bound anti-CD3 and human recombinant ICOS ligand chimera, in the absence (Neg Ctl) or presence of DCM (Ctl, EBV dUTPase, or HHV6A dUTPase) alone or together with IL-12. Treatment of cells with activin A plus IL-12 was used as the positive control, and cells stimulated with beads only were used as the negative control. Data represent the mean ± SEM of *n* = 6–8 (**A**, **C**, and **D**) and *n* = 4 (**B**) independent experiments. *P < 0.05, **P < 0.01, ***P < 0.001, ****P < 0.0001 of treatments versus negative Ctl or beads by 1-way ANOVA Kruskal-Wallis multiple comparisons test with Dunn’s correction.

We next examined whether DCM from EBV and HHV-6A dUTPase protein–treated hDCs could induce naive CD4^+^ T cells to differentiate into IL-21–producing cells. For this, naive CD4^+^ T cells were stimulated for 3 days with anti-CD3/anti-CD28–coated beads alone or in the absence (Ctl DCM) or presence of EBV dUTPase DCM (25% vol/vol). On day 3, cells were restimulated with phorbol 12-myristate 13-acetate plus ionomycin (PMA/I) or left unstimulated, and IL-21 levels in culture supernatants were determined by ELISA. As shown in Figure 5A, treatment of activated naive CD4^+^ T cells with EBV dUTPase DCM resulted in a significant 4.8-fold increased secretion of IL-21 (504.8 ± 0.315 pg/mL) relative to the levels induced by Ctl DCM (104.8 ± 18.3 pg/mL, *P* = 0.0291) or by beads plus restimulation on day 3 with PMA/I, (133 ± 1.73 pg/mL, *P* = 0.0021). A 2-fold increase in IL-21 levels was also observed in supernatants of cells treated with EBV dUTPase DCM followed by restimulation with PMA/I compared with Ctl DCM plus PMA/I restimulation (1719 ± 37.8 pg/mL versus 822.9 ± 0.285, *P* = 0.0268). Examination of IFN-γ levels in the supernatants of naive CD4^+^ T cells differentiated as described above for 3 days revealed the presence of heightened IFN-γ concentrations in supernatants of cells expressing a T_FH_-like marker signature, suggesting that these cells exhibit a T_FH1_ phenotype (Figure 5B).

To determine whether EBV dUTPase DCM had the ability to regulate T_FH_ cell function in vitro, we measured the production of CXCL13, a chemokine exclusively secreted by GC T_FH_ cells after cognate interactions with costimulatory molecules on the surface of B cells. Examination of CXCL13 levels in supernatants of naive CD4^+^ T cells stimulated for 5 days with plate-bound anti-CD3 and human recombinant ICOS ligand chimera in the absence or presence of EBV dUTPase DCM resulted in a 2.4-fold increased secretion of CXCL13 induced by EBV dUTPase DCM (149.14 ± 11.02 pg/mL) compared with the unstimulated control (62.53 ± 2.78 pg/mL, *P* ≤ 0.0095) (Figure 5C). CXCL13 secretion induced by EBV dUTPase DCM was enhanced by IL-12 (175.22 ± 11.31 pg/mL). Furthermore, a significant increase in IL-10 levels was also observed in supernatants of activated naive CD4^+^ T cells treated with EBV/HHV-6A dUTPase DCM compared with cells stimulated with Ctl DCM (135.85 ± 24.68 pg/mL and 83.82 ± 6.42 versus 33.77 ± 1.38, for EBV dUTPase, HHV-6A dUTPase, and Ctl DCM, respectively, *P* ≤ 0.0286) (Figure 5D). IL-10 is known to provide key survival signals to B cells and to promote Ab class-switching.

**Figure 6:**
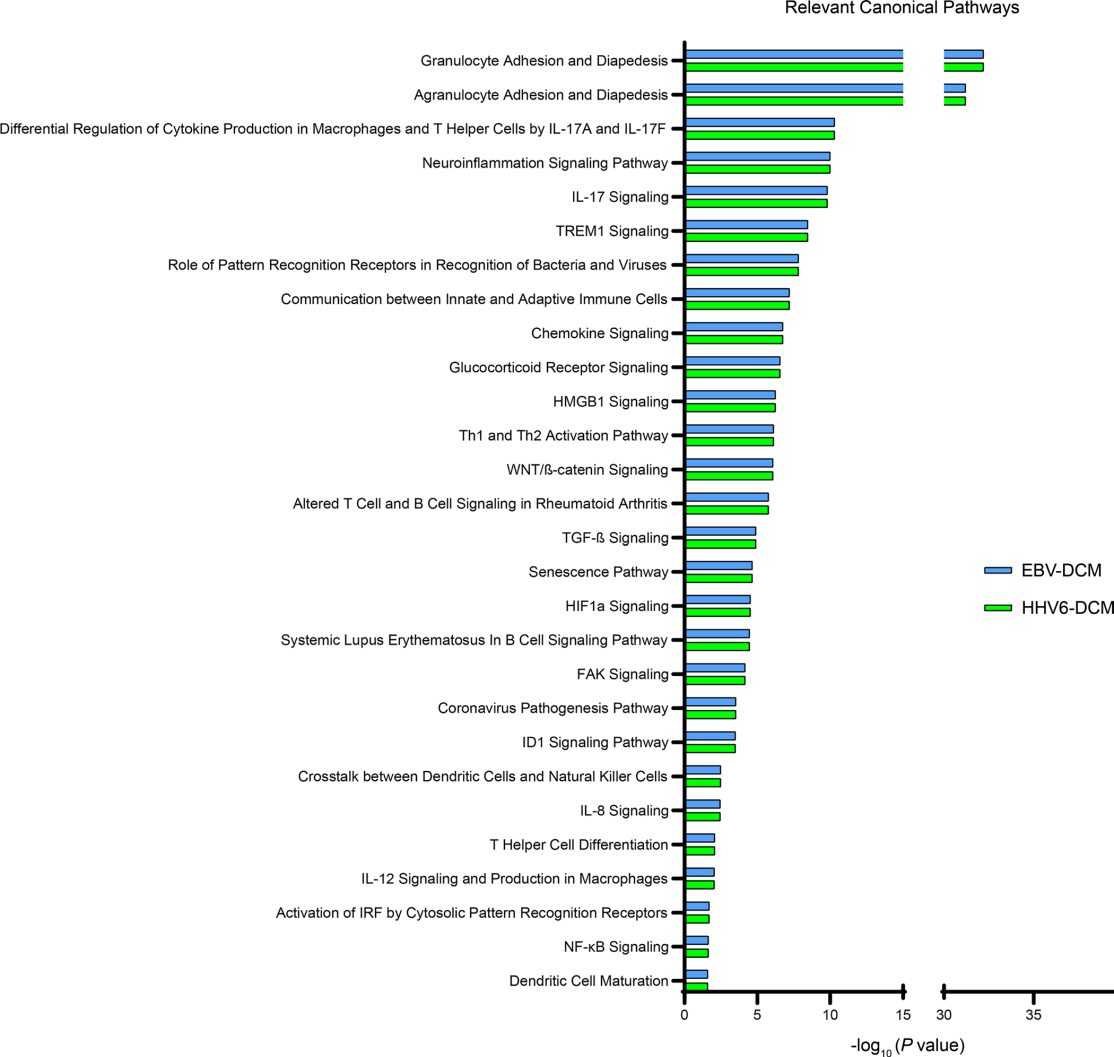
EBV and HHV-6A dUTPase DCM are enriched for proteins involved in immune cell activation and function pathways. Monocyte-derived hDCs (2.5 × 10^5^ cells) were treated with dUTPase protein (10 μg/mL) from HHV-6A or EBV or left untreated (control) for 24 hours. After treatments, culture supernatants were characterized using the Human L-1000 Antibody Array (RayBiotech), as described in Methods. Differentially expressed proteins within the EBV and/or HHV-6A dUTPase DCM, compared with Ctl DCM, were further analyzed using the Core Analysis tool within the Ingenuity Pathway Analysis software to determine associated biological pathways. Bar graphs represent significance of pathway association with differentially expressed proteins from EBV (blue) or HHV-6A (green) dUTPase DCM. All pathways displayed had log(*P*) > 1.3, by right-tailed Fisher’s exact test with Benjamini-Hochberg method correction and –log_10_ transformation.

To better understand the effects of EBV and HHV-6A dUTPases on hDC function and downstream implications in T_FH_ cell differentiation, we performed a comprehensive analysis of culture supernatants from hDCs stimulated for 24 hours with recombinant EBV and HHV-6A dUTPase proteins (10 μg/mL) or unstimulated hDCs for the presence of additional key factors important for T cell activation and T_FH_ cell differentiation using a commercial human Ab array. We found that EBV and HHV-6A dUTPases differentially induced the protein expression/secretion of various B and T cell chemoattractants as well as proteins involved in the regulation of B and T cell differentiation and function as well as GC development and maintenance (Tables 2 and 3). For instance, production of the activin A receptor RIIA, as well as the proteins CCL27; CCR7; CXCL11; CXCL16; CXCR2; follistatin; frizzled 1, 4, and 7; and MMP-9 and -10, were induced by both EBV and HHV-6A dUTPases at similar levels. However, eotaxin-2, frizzled 3 and 5, I-309/CCL1, IL-22, IL-31RA, IP-10, leptin, MCP-1 and -2, MIP-1A, OX40L, SMAD5 and 7, TARC, TECK/CCL25, TGF-α and -β, TSLPR, and TSLP were induced more strongly by EBV dUTPase. Heightened protein levels of DTK, GRO/CXCL1, IL-6, IL-8, M-CSF, MIP-1β, MIP-2, and MMP-1 were induced by HHV-6A dUTPase. Follow-up analysis of differentially expressed proteins induced by EBV and HHV-6A dUTPases using the Core Analysis tool within Ingenuity Pathway Analysis software revealed that the dUTPases significantly associated with recognition of viruses through pattern recognition receptors (–log_10_*P* = 7.82), T helper cell differentiation (–log_10_*P* = 2.08), IL-12 signaling/production in macrophages (–log_10_*P* = 2.05), NF-κB signaling (–log_10_*P* = 1.65), and DC maturation (–log_10_*P* = 1.6), among others involved with immune activation and function (Figure 6).

### EBV dUTPase injections induce T_FH_ cell differentiation in spleens of C57BL/6 female mice.

**Figure 7:**
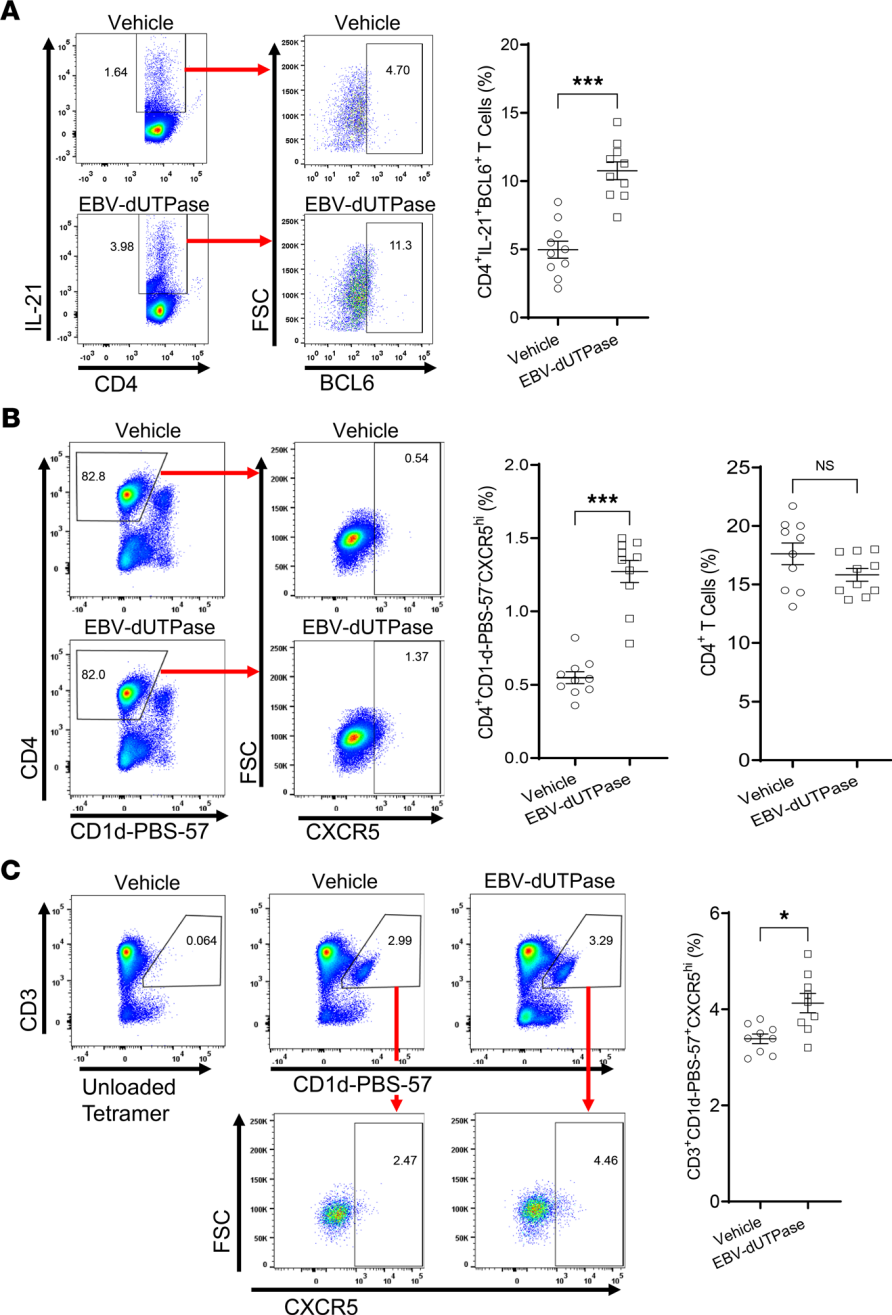
EBV dUTPase injections induce T_FH_ cell differentiation and the formation of NKT_FH_ cells in spleens of C57BL/6 female mice. (**A**) Spleen single-cell suspensions (splenocytes) from EBV dUTPase– or vehicle-injected mice for 5 consecutive days were prepared as described in Methods. Cells were immediately stained with Abs against CD4, IL-21, BCL6, CD19 (B cells), CD11b (monocytes), and CD11c (DC), and the expression of T_FH_ markers IL-21 and BCL6 was examined by flow cytometry analysis. Flow cytometry analysis was conducted on the lymphocyte population and pregated on live (FVD^–^) CD19^–^CD11b^–^CD11c^–^ cells. Representative flow cytometry plots indicating frequency (%) of CD4^+^IL-21^+^ cells in gate (left) or BCL6^+^ T cells (right) in EBV dUTPase– and vehicle control–injected mice. (**B** and **C**) Splenocytes (prepared following injections as in **A**) were depleted of CD19^+^ B cells and CD8^+^ T cells by magnetic cell separation using MojoSort nanobeads, as described in Methods. Enriched cells were stained with Abs against B220, CD11b, CD11c, CD4, CD3, CD1d-PBS-57 tetramer, and CXCR5, and the expression of tetramer subsets was analyzed by flow cytometry. Flow cytometry samples were pregated on live (FVD^–^) B220^–^CD11b^–^CD11c^–^ cells. (**B**) Flow cytometry of splenic CD4^+^ CD1d-PBS-57 tetramer–negative T cells (gating), indicating percentage of CXCR5^+^ T_FH_ cells. (**C**) Flow cytometry and relative frequencies of CD1d-PBS-57 tetramer–positive CD3^int^ NKT cells in gate (left plots) or CXCR5^hi^ NKT_FH_ cells (right plots). Bar graph represents quantitative data of NKT_FH_ cells in mice. Data represent the mean ± SEM of 3 independent experiments; *n* = 20 (10 mice/group) (**A**), *n* = 10 (5 mice/group) (**B**). *P < 0.05, ***P < 0.001, by 2-tailed Mann-Whitney *U* test (**A**–**C**).

To investigate whether the T_FH_ cell differentiation effects of EBV dUTPase observed in vitro also occurred in vivo, mice were injected daily with dUTPase protein or vehicle control, 5 days later spleen cells were isolated, and their phenotypes were examined by flow cytometry. Analysis of splenic CD4^+^ T cell populations identified a significantly increased number of cells expressing the characteristic T_FH_ cell markers IL-21, BCL6 (Figure 7A), and CXCR5 and not binding to tetramers of PBS-57–loaded CD1d (Figure 7B) in mice injected with EBV dUTPase compared with mice that received vehicle. While no changes were observed in the percentage of total CD4^+^ T cells (Figure 7B, far right graph). A second cell population was identified as being CD4^+^BCL6^–^IL-21^+^CXCR5^+^ (0.846 ± 0.0252% of total CD4^+^ T versus 0.458 ± 0.111%, *P* = 0.0092 by 2-tailed Student’s *t* test), which represents a CD4^+^ pre-T_FH_ cell precursor. A subset of NKT cells with follicular helper properties (NKT_FH_), defined as CD3-intermediate (CD3^int^), binding to CD1d-PBS-57–loaded tetramers and expressing high levels of CXCR5, was significantly increased in mice injected with EBV dUTPase compared with mice receiving vehicle (Figure 7C).

### EBV dUTPase protein promotes MZ B, GC B, and plasmablasts/plasma cells’ formation.

**Figure 8:**
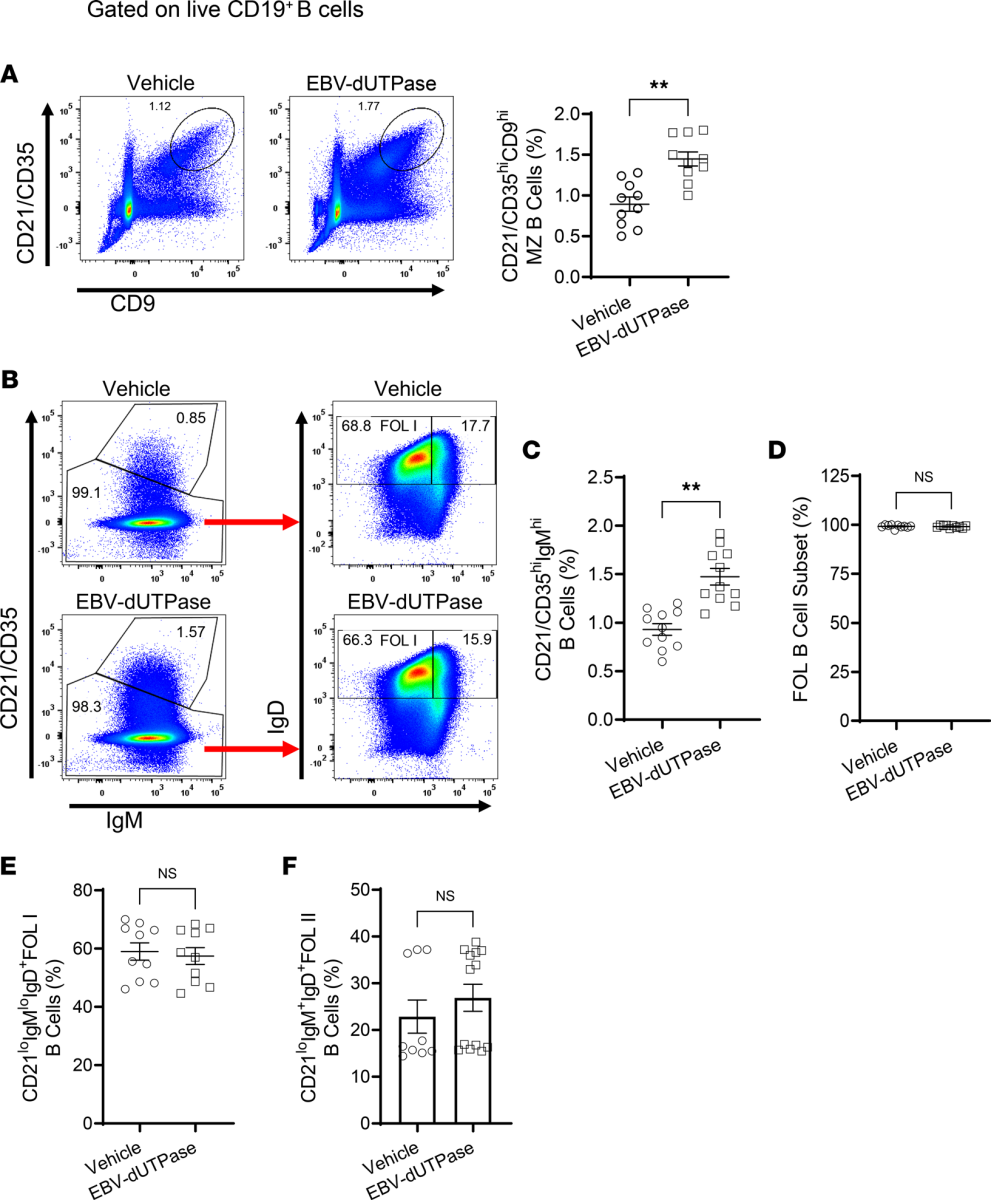
EBV dUTPase induces the formation of MZ B cells in spleens of C57BL/6 female mice. Splenocytes from EBV dUTPase or vehicle injected mice for 5 consecutive days were prepared and mouse B cell subsets isolated as described in Methods. Representative flow cytometry plots and quantitative bar graphs of (**A**) MZ B cells (CD19^+^CD21/CD35^hi^CD9^hi^, (**B**) MZ B cell subset (CD21^hi^IgM^hi^) (left plots, top box), follicular (FOL) B cell subset (left plots, bottom box), FOL I B cells (CD21^lo^IgM^lo^IgD^+^), and FOL II B cells (CD21^lo^IgM^+^IgD^+^) (left plots) in mice injected daily with EBV dUTPase or vehicle for 5 days. (**C**–**F**) Quantitative bar graphs of data in **B**. Data represent the mean ± SEM of 3 independent experiments, *n* = 20 (10 mice/group). **P < 0.01, by 2-tailed Mann-Whitney *U* test.

**Figure 9:**
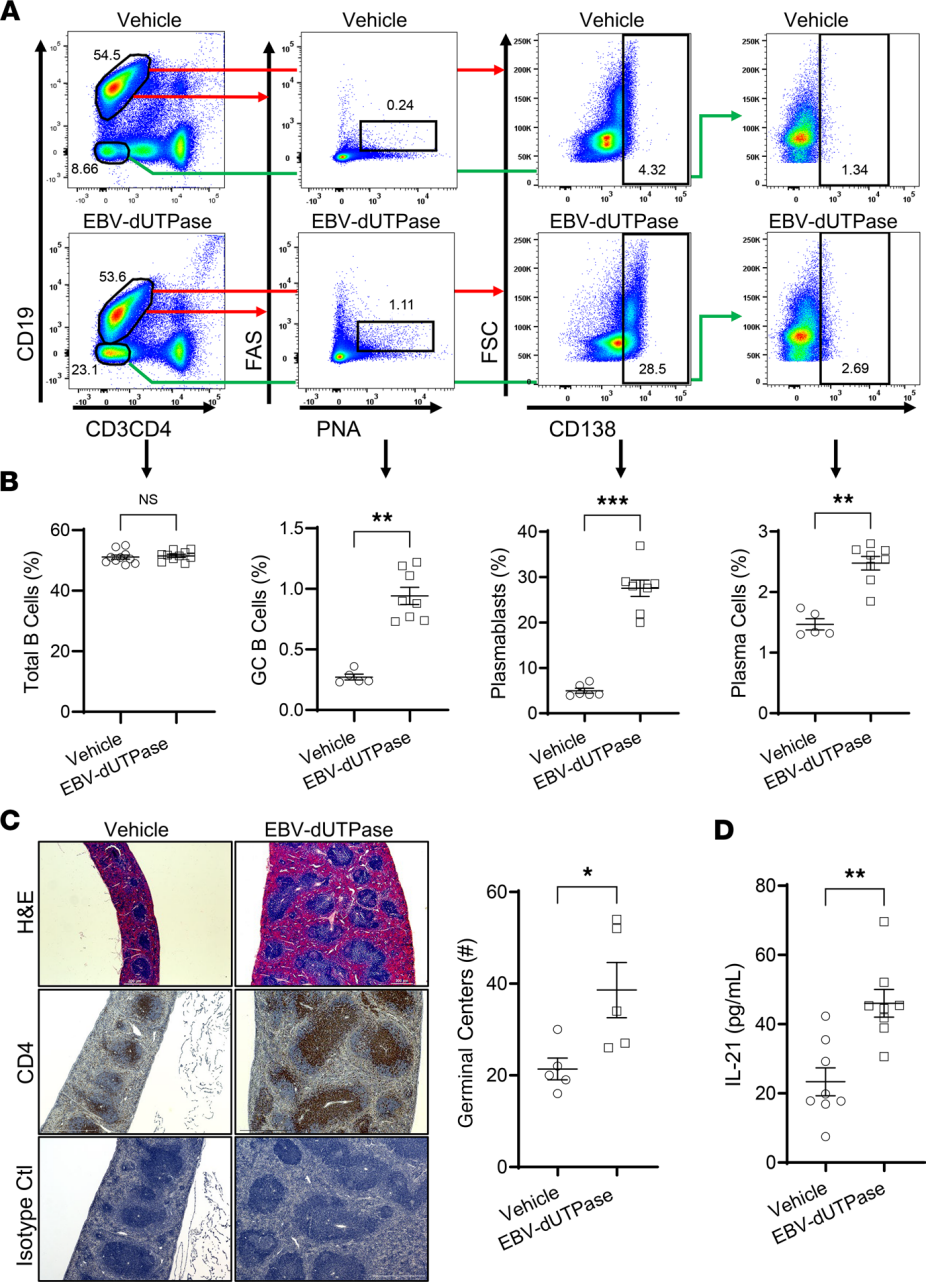
EBV dUTPase increases GC B cell frequencies in vivo. (**A**) Representative flow cytometry plots of splenocytes from vehicle-injected (top plots) or EBV dUTPase–injected (bottom plots) mice (for 6 days) and stained with Abs against CD19, CD3, CD4, FAS, peanut agglutinin (PNA), and CD138. Values in the plots indicate the percentage of GC B cells (FAS^+^PNA^+^ — plots), plasmablasts (CD19^+^CD138^+^ — center plots), or plasma cells (CD19^–^CD138^+^ — far right plots). (**B**) Quantitative graphs of **A** indicating total B cells, GC B cells, plasmablasts, and plasma cells. (**C**) Staining of formalin-fixed, paraffin-embedded spleen sections from C57BL/6 mice injected as in **A**. H&E staining of spleen sections from vehicle- or EBV dUTPase–injected mice showing spleen size (top images), and number of GC differences (quantitative graph) between the 2 groups (31 GCs in EBV dUTPase versus 20 in vehicle, *P* < 0.0455 by 2-tailed Mann-Whitney test). Immunohistochemical staining of splenic CD4^+^ T cells (middle images). Isotype control Ab (bottom images) was used as a negative control. Original objective magnification: 5×, 300 μm scale bar (H&E top images); 10×, 500 μm scale bar (middle and bottom images). (**D**) ELISA of IL-21 in whole spleen lysates from EBV dUTPase– or vehicle control–injected mice. Data represent the mean ± SEM of 3 experiments, *n* = 16 (8 mice/group) (**A** and **D**); 2 experiments, *n* = 10 (5 mice/group) (**C**). *P < 0.05, **P = 0.008, ***P < 0.001, by 2-tailed Mann-Whitney *U* test.

Immunophenotyping studies by flow cytometry of splenic pan-B cells isolated by magnetic separation (negative selection) from mice injected daily with EBV dUTPase protein or vehicle for 5 days demonstrated that EBV dUTPase protein induced a significant increase in the frequency of MZ B cells defined as CD19^+^CD21/CD35^hi^CD9^hi^ (Figure 8A) as well as in the frequency of the MZ B cell subset, which includes MZ B and MZ precursors and is defined as CD19^+^CD21/CD35^hi^IgM^hi^ (Figure 8B, top box on left plots, and Figure 8C). Analysis of the FOL B cell subset revealed no significant differences in FOL I (CD21/CD35^lo^IgM^lo^IgD^+^) or FOL II (CD21/CD35^lo^IgM^+^IgD^+^) populations between EBV dUTPase protein–injected and control mice (Figure 8B, left plots; and Figure 8, D–F). We next investigated whether EBV dUTPase could affect GC formation/development in the spleen, a key site of autoantibody production. Flow cytometric analysis of splenocytes costained with peanut agglutinin (PNA) and FAS showed that the dUTPase protein increased GC B cell frequencies following i.p. injections for 6 days (Figure 9, A and B). An increase in the frequencies of splenic plasmablasts and plasma cells defined as CD19^+^CD138^+^ and CD19^–^CD138^+^, respectively, was also observed in the spleens of mice injected with EBV dUTPase compared with mice receiving vehicle (Figure 9, A and B). Furthermore, H&E staining of formalin-fixed, paraffin-embedded spleen sections from EBV dUTPase– or vehicle-injected C57BL/6 mice (10 mice; 5 mice/group) for 6 days showed increased spleen size and a significantly higher number of GCs in EBV dUTPase–treated mice (31 GCs) relative to the controls (20 GCs) (Figure 9C, top images and quantitative dot plot graph). Immunohistochemical examination of spleen sections revealed an increased presence of CD4^+^ T cells within the GC in spleen sections of EBV dUTPase–treated mice compared with vehicle controls (Figure 9C, middle images). The isotype control Ab, used as a negative control, did not detect expression of CD4 (Figure 9C, bottom images).

Further ELISA analysis of spleen lysates demonstrated a significant increase in the concentration of IL-21 in the spleens of mice (*n* = 9) injected with EBV dUTPase protein compared with vehicle controls (42.9 ± 4.7 pg/mL versus 24.2 ± 4.6 pg/mL, *P* = 0.0145) (Figure 9D).

**Figure 10:**
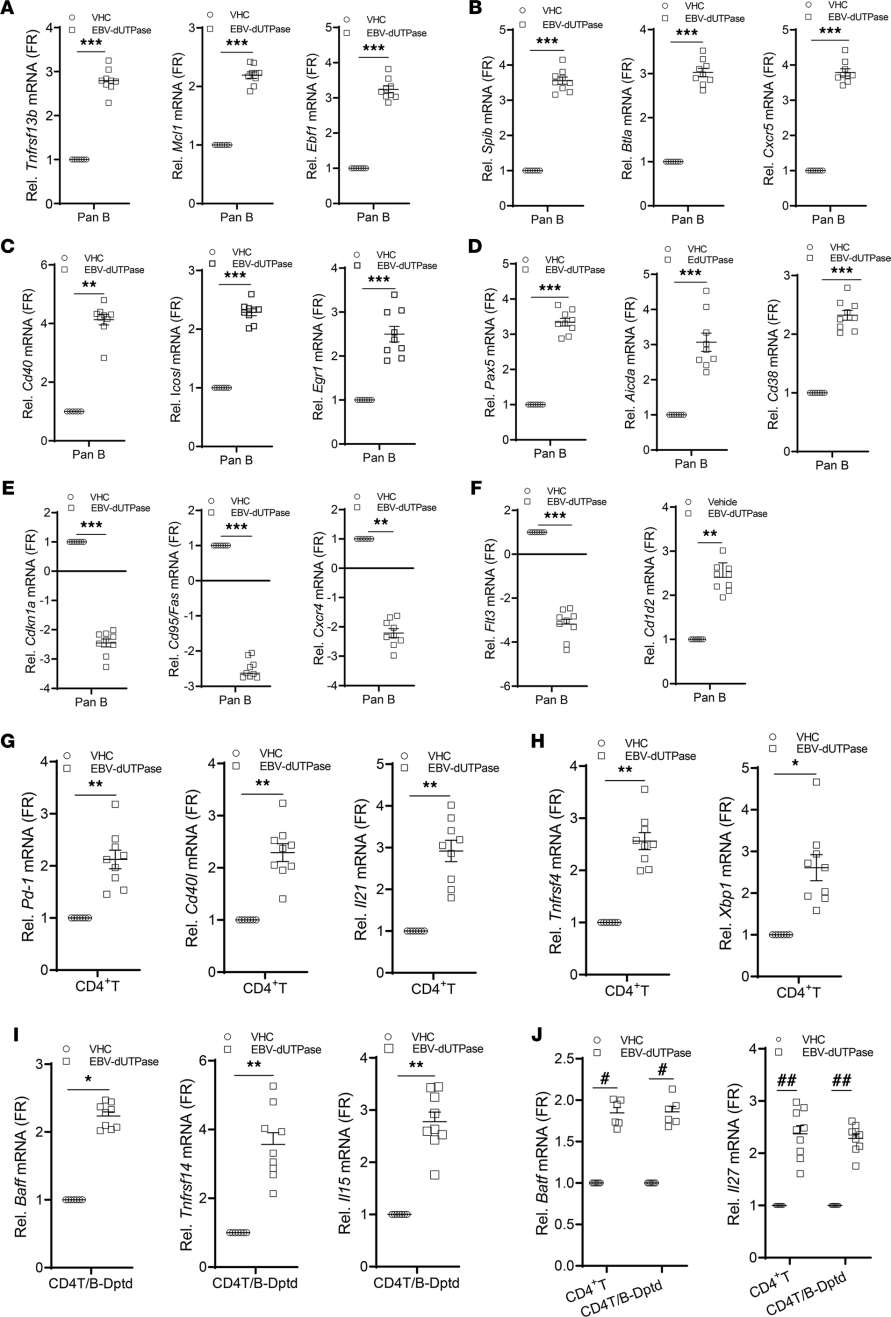
EBV dUTPase induces extrafollicular, GC B, and T_FH_ transcriptional gene expression programs in vivo. Quantitative RT-PCR expression analysis of select gene signatures in splenic (**A**–**F**) pan-B cells and (**G**–**J**) CD4^+^ T/NKT-enriched cells or cells depleted of pan-B and CD4^+^ T/NKT isolated by magnetic separation from spleen single-cell suspensions of EBV dUTPase– or vehicle-injected mice for 6 days. Data were normalized to *B2m* and *Hsp90ab1* and expressed as fold regulation relative to the vehicle control group. For all panels, data represent the mean ± SEM of *n* ≥ 8 individual data points *n* = 16 (8 mice/group), 2 experiments. *P = 0.0295, **P < 0.008, ***P = 0.0006 of dUTPase versus vehicle control treatment by 2-tailed Mann-Whitney *U* test (**A**–**I**) or ^#^*P* < 0.05, ^##^*P* < 0.01 by Kruskal Wallis multiple-comparison test (**J**).

Initial gene expression analyses of splenocytes isolated from mice injected with either the EBV dUTPase protein or vehicle for 5 days demonstrated that the dUTPase protein modulated the expression of several genes (≥2-fold), whose products are involved in the differentiation and function of immune cells (IL-7, IL-11, CSF-2), T cell activation and proliferation (CD28), Ab production/regulation (RAG1, recombination activating gene 1; TNFRSF14, herpesvirus entry mediator), as well as induction of proinflammatory cytokines (IL-1β, IL-6, and SPP1, osteopontin). Notably, the genes encoding IL-7 and IL-11, which are involved with B and T cell development, V-D-J rearrangement of TCRβ, and T cell–dependent development of Ig-producing B cells, were significantly downregulated by 53- and 2.4-fold, respectively. We further investigated the effect of EBV dUTPase protein on the expression of T_FH_ genes as well as genes involved with B cell activation/differentiation and GC development, in 3 splenic cell populations (pan-B–enriched cells, CD4^+^ T/NKT-enriched cells, and pan-B + CD4^+^ T/NKT–depleted cells) isolated from mice injected daily with dUTPase protein or vehicle for 6 days. Quantitative real-time PCR (QRT-PCR) analysis of transcripts in pan-B–enriched cells revealed that EBV dUTPase increased the mRNA expression of *Tnfrsf13b/Baff*, *Mcl1* (myeloid cell leukemia 1), *Ebf1* (early B factor 1), *Spib*, *Btla* (B and T lymphocyte attenuator), *Cxcr5* (Figure 10, A and B), *Cd40*, *Icosl*, the transcription factors *Egr1* (early growth response 1) and *Pax5* as well as *Aicda* (activation induced cytidine deaminase), and the cell activation marker *Cd38* (Figure 10, C and D), while downregulating the cell cycle inhibitor *Cdkn1a*, *Fas* (cell dead receptor), *Cxcr4*, and *Flt3* mRNA expression (Figure 10, E and F). Interestingly, EGR-1 is an immediate-early transcription factor that is required for entry of EBV-infected B cells into the lytic cycle. EBV dUTPase protein also increased *Cd1d2* mRNA expression (2.47 ± 0.25; Figure 10F), which encodes an MHCI-like molecule involved with presentation of lipid antigens to T cells and activation of NKT cells. Similarly, and consistent with the human in vitro T_FH_ differentiation data (Figures 4 and 5), EBV dUTPase induced the upregulation of *Pd-1*, *Cd40l*, *Il21*, *Ox40/Tnfrsf4*, and *Xbp1* mRNA expression in CD4^+^ T/NKT-enriched cells (Figure 10, G and H). However, in pan-B + CD4^+^ T/NKT–depleted cells, the dUTPase induced the expression of *Tnfsf13b/Baff*, *Tnfrsf14/Hvem*, and *Il15* (Figure 10I). Interestingly, activation of OX40 signaling in T_FH_ cells has been shown to play a pathogenic role in murine lupus and human systemic lupus erythematosus. Increased basic leucine zipper transcription factor ATF-like (*Batf*) and *Il27* mRNA levels were observed in both CD4^+^ T/NKT-enriched and pan-B + CD4^+^ T/NKT–depleted cell fractions (Figure 10J, far right graphs). BATF is a positive regulator of T_FH_ cells with proallergic function while IL-27 promotes the survival of IL-21–producing T_FH_ cells.

## Discussion

ME/CFS is a chronic, multisystem illness of unknown etiology. The case definition recently established by the Institute of Medicine (3) requires that a patient have a substantial reduction or impairment in the ability to engage in pre-illness activities, unrefreshing sleep, post-exertional malaise, and either cognitive impairment or orthostatic intolerance. However, these patients exhibit a wide spectrum of symptoms, including persistent or relapsing unexplained fatigue of at least 6 months’ duration; headaches; myalgias; arthralgias; sore throats; lymphadenopathy; hypersensitivity to noise, light, or certain foods; and autonomic disturbances (2). Since no biomarkers are available to aid in the diagnosis of ME/CFS, diagnosis is usually based upon exclusion of other syndromes and diseases. This results in a very heterogeneous patient population and severely complicates the ability to elucidate mechanisms that contribute to the onset and progression of this disease, especially considering the possibility of multiple etiologies.

This study demonstrates for the first time to our knowledge the presence of heightened serum levels of activin A and IL-21 in a subgroup of individuals diagnosed with ME/CFS, which positively correlated with seropositivity for anti-EBV and anti-HHV6A dUTPase Abs in these patients. Notably, both EBV and HHV-6A dUTPases proteins are strong inducers of activin A secretion in hDCs, further supporting a role of these herpesviruses’ proteins in the pathophysiology of ME/CFS. Activin A belongs to the TGF-β superfamily and is a pleiotropic cytokine affecting several cell types involved with immune regulation. Activin A was recently identified as a potent regulator of T_FH_ cell differentiation in humans (19) and has also been implicated in several autoimmune and inflammatory diseases, although a causal effect has not been established (22). Elevated activin A expression has been linked to muscle wasting and loss of muscle mass (23, 24), which may contribute to the fatigue observed in ME/CFS. Furthermore, several studies have reported decreased NK cell function in ME/CFS patients (25–27), and activin A has been shown to attenuate NK cell function in humans (28) and mice (29).

Our in vitro studies using ME/CFS serum with elevated activin A levels as well as virus dUTPase–derived conditioned media showed differential induction of a T_FH_ cell–like phenotype characterized by high expression of PD-1, ICOS, and CXCR5 and secretion of IL-21, in an activin A–dependent mechanism. To the best of our knowledge, this is the first study reporting the presence of T_FH_ cell regulators in the serum of ME/CFS patients that are capable of modulating T_FH_ cell differentiation in vitro. Likewise, IL-21 is a pleiotropic cytokine, with broad-range effects on immune and nonimmune cells, produced primarily by invariant NKT (iNKT) cells, T_FH_ cells, and T_H17_ cells (29). It is required for the differentiation of T_FH_ cells, which are important for the GC Ab response and are key regulators of humoral immunity. More importantly, increased serum IL-21 levels have been reported in patients with autoimmune diseases, including systemic lupus erythematosus, rheumatoid arthritis, multiple sclerosis, and Sjögren’s syndrome (30), diseases that have been linked to EBV infection.

Somewhat striking was the finding that only a small number of ME/CFS cases’ sera (*n* = 14; 3.77%) exhibited increased CXCL13 levels above baseline despite the presence of high levels of IL-21. Serum/plasma CXCL13 is a marker of T_FH_ cell function and represents total GC activity (31). Notably, the lack of simultaneous increase in CXCL13 in ME/CFS cases implies that a subgroup of patients with ME/CFS producing excessive levels of activin A, IL-21, or a combination thereof and abnormally low CXCL13 levels exhibit defects in the normal GC reaction/Ab response, which may contribute to the immune dysfunction observed in these patients.

Our findings concerning serum activin A levels are in conflict with the study by Lidbury et al. (32), who reported a significant increase in activin B but not activin A. The reasons for these differences are unknown but may reflect differences in the genetic background of these individuals and/or variations in the case definition criteria used. However, a recent study by the same group using a different cohort of patients with ME/CFS (*n* = 134) reported no significant differences in activin B plasma levels between ME/CFS cases and controls (*n* = 54) (33). Thus, using activin B levels as a biomarker for ME/CFS remains controversial.

In line with a recent study that reported the presence of a significant increase in T_FH_ subsets in patients with infectious mononucleosis, a disease caused by EBV (17, 18), we demonstrate that EBV dUTPase and HHV-6A dUTPase–derived DCM, which contained high concentrations of activin A, also promoted the differentiation of naive CD4^+^ T cells into a T_FH_ cell–like phenotype. More importantly, the differentiated T_FH_ cells were also able to provide B cell help in vitro as determined by the increased secretion of CXCL13. In vivo follow-up studies in mice demonstrated that the EBV dUTPase protein induced the formation of T_FH_ cells. The induction of activin A by the dUTPase protein released during EBV pre-latent phase (34) would result in the differentiation and proliferation of T_FH_ cells, which are required for the GC response and the establishment and maintenance of EBV latency. While studies to address the modulation of the GC reaction by HHV-6A/B are lacking, a recent study showed reactivation of HHV-6B in follicular DCs in a small number of patients with chronic/recurrent lymphadenopathy, suggesting that HHV-6A/B may modulate the lymph node microenvironment (35).

In addition to altering the GC Ab response, the present study shows that EBV dUTPase protein is stimulating an extrafollicular Ab response, as determined by the significant increase in the frequency of iNKT_FH_ cells, MZ B cells, and plasmablasts/plasma cells observed in vivo in mice injected with EBV dUTPase protein. These findings are supported by gene expression analyses of splenic pan-B and CD4^+^ T populations as well as a previous study (13). iNKT cells can provide B cell help in a cognate T cell–dependent response (36–38). Such an interaction leads to the development of extrafollicular foci, abortive GC formation, low-affinity maturation, and short-lived plasma cells. MZ B cells also differentiate into short-lived, low-affinity extrafollicular plasma cells, resulting in a rapid Ab response to pathogens (39, 40). If dysregulated, these processes can lead to increased production of autoreactive Abs and autoimmunity.

Studies to address B cell populations in patients with ME/CFS have provided conflicting information due in part to the small number of patients examined in each study and possibly due to the heterogeneity of these populations (41–43). A single study indicated no differences in MZ B cells between patient and control groups (42). Conversely, several studies have shown an increase in iNKT cells in patients with ME/CFS (44–46), but no differences were found in the levels of perforin, granzyme A and B, or CD57 relative to the control cohort (45). Furthermore, the frequency of iNKT cells positively correlates with disease severity (44, 45). However, additional studies are needed to determine the functional implications of having increased iNKT frequencies in the context of cognate interactions with MZ B cells and disease outcome.

Our studies demonstrate that the EBV and HHV-6A dUTPase proteins are strong inducers of activin A in hDCs and can drive T_FH_ differentiation both in vitro and in vivo. In vivo studies of EBV dUTPase also revealed a downregulation of *Cxcr4*, a gene coding for a chemokine receptor important for B cell trafficking back to the dark zone (47). Altogether, these data suggest that some patients with ME/CFS may exhibit a dysfunctional GC Ab response. Furthermore, our results support a role for EBV dUTPase protein in this process by stimulating an extrafollicular Ab response as evidenced by the observed increase in splenic iNKT_FH_ and MZ B cells, which could result in the formation of autoreactive B cells and, subsequently, the production of autoreactive Abs. We are proposing that in a subset of patients with ME/CFS there is increased abortive lytic replication of EBV (8) and possibly HHV-6, especially in those patients exhibiting a diminished EBV-specific B and T cell response (48), resulting in the increased release of EBV dUTPase possibly in exosomes (13). The release of virus dUTPase protein in the cellular microenvironment could result in chronic stimulation of the host immune system, potentially impairing the extrafollicular and GC Ab responses. These studies also confirm that patients diagnosed with ME/CFS are extremely heterogeneous as evidenced by the extreme differences observed between serum concentrations of activin A and IL-21 among patients. Additional studies are required to determine the relationship, if any, between levels of activin A, IL-21, or the combination of the two with symptomology and disease severity. The results also suggest that a combination screening to assess serum levels of EBV/HHV-6A anti-dUTPase Ab, activin A, and IL-21 followed by immunophenotyping studies to elucidate T_FH_ and iNKT_FH_ cell frequencies may be useful not only as biomarker signatures to identify patients with ME/CFS but also to aid in subgrouping or stratifying patients. Such an approach may be useful in developing novel therapeutic approaches, as well as personalized medicine for patients with ME/CFS. Finally, these data raise awareness of the fact that the presence of increased Abs against the herpesviruses EBV/HHV-6A dUTPases, above baseline levels, which normally occurs even in the absence of productive lytic reactivation, may contribute to the symptomology observed in a subset of ME/CFS cases.

## Methods

### Study participants, informed consent, and ethical review.

All human serum samples used in this study were deidentified and approved by the Institutional Review Board (IRB) of The Ohio State University. Human blood sample collection and experimental procedures were approved by the IRB committee at Nova Southeastern University. Written informed consent was obtained from all participants in accordance with the Declaration of Helsinki. Study participants included 351 with ME/CFS, 54 with GWI, and 77 healthy controls from previously published studies (8, 49). Demographics of study cohorts are provided in Table 1. Further detailed information regarding inclusion/exclusion criteria for the recruitment of participants has been described elsewhere (8, 49, 50).

### Human cytokine ELISAs.

Serum levels of activin A, IL-21, and CXCL13 in patients with ME/CFS were determined by ELISA following the manufacturer recommendations. Cytokine/chemokine concentrations were determined using the human activin A ELISA kit (EHACTIVINA, Thermo Fisher Scientific), IL-21 ELISA (ELH-IL21, RayBiotech), and CXCL13/BLC/BCA-1 Quantikine ELISA (DCX130, R&D Systems, Bio-Techne). The minimum detectable dose of activin A, IL-21, and CXCL13 was determined to be 15 pg/mL, 110 pg/mL, and 1.64 pg/mL, respectively. Minimum detectable dose is defined as the analyte concentration resulting in an absorbance that is 2 SD higher than that of the blank (diluent buffer). Concentrations are expressed as pg/mL and represent the mean ± SEM. Normal serum levels for these cytokines, based on literature reports, are 1000 pg/mL (activin A), 115 pg/mL (IL-21), and 126 pg/mL (CXCL13).

### Mouse IL-21 ELISA.

Spleens were homogenized in 1 mL of ice-cold PBS containing 1× complete protease inhibitor cocktail (Roche Applied Science), using an Omni International Bead Ruptor 12 instrument (PerkinElmer) for 20 seconds at high speed followed by a centrifugation step at 12,000*g* for 10 minutes at 4^o^C. Protein concentration was determined by Bio-Rad protein assay. IL-21 levels were determined using a mouse IL-21 ELISA kit (R&D Systems, Bio-Techne), normalized to the protein concentration and expressed as pg/mg protein. Minimum detectable concentration was 62 pg/mL.

### Purification of recombinant dUTPases from EBV and HHV-6A.

Subcloning and purification of recombinant dUTPase proteins were performed as previously described (11, 15, 16). All recombinant dUTPase protein preparations were tested for the presence of contaminants and were free of detectable levels of LPS, peptidoglycan (SLP-HS), DNA, or RNA. Protein concentration was determined using the Qubit fluorimeter and stored at −80°C.

### dUTPase ELISAs.

Herpesvirus dUTPase ELISAs were performed as described previously (8). Briefly, Nunc-Immuno Plate MaxiSorp 96-well plates (MilliporeSigma) were coated overnight at 4°C with recombinant dUTPase protein at 2.5 μg/mL in PBS. All serum samples were used at a 1:800 dilution in blocking buffer and incubated for 2 hours at room temperature (RT). Plates were washed 3 times with PBS/0.05% Tween 20 followed by incubation with anti-human IgG horseradish peroxidase–conjugated (HRP-conjugated) secondary Ab (catalog A0170, MilliporeSigma) at 1:1000 dilution for 1 hour. Plates were washed 6 times and incubated for 15 minutes with 100 μL of OPD substrate (Invitrogen, Thermo Fisher Scientific). Sulfuric acid (50 μL of 2 M H_2_SO_4_) was added to stop the reaction, and plates were read at 490 and 690 nm on a Lab Systems Multiskan MCC/340 plate reader using the Genesis v3.05 Life Sciences Ltd software. The background from the 490 nm uncoated wells and PBS-BSA (negative controls) was subtracted from the mean absorbance of the coated wells. A positive reaction was defined as a serum sample that led to a signal 3 times over the background OD of the control serum. All clinical serum/plasma samples were run at least in duplicate.

### Cell culture.

Monocyte-derived hDCs and human PBMCs from healthy controls (*n* = 5) were obtained from Astarte Biologics. Cells (hDCs) were maintained in X-VIVO 15 serum-free medium (Lonza) supplemented with 500 U/mL GM-CSF and IL-4.

### hDC treatments.

Cells were seeded at a density of 2.5 × 10^5^ in 24-well plates and cultured in X-VIVO 15 serum-free medium supplemented with 500 U/mL GM-CSF and IL-4. The next day, hDCs were stimulated with dUTPase protein (10 μg/mL) from EBV, HHV-6A, and human dUTPase (in some experiments) alone or left untreated for 24 hours, as described previously (12–14). Following treatments, culture supernatants (DCM) were collected for determination of activin A levels by ELISA and/or for in vitro treatments of naive CD4^+^ T cells as described below.

### Human naive CD4^+^ T cell treatments.

Naive CD4^+^ T cells were isolated from PBMCs by magnetic bead negative selection using the EasySep Human Naïve CD4+ T Cell Isolation Kit (STEMCELL Technologies). Cell purity (CD4^+^CD45RA^+^CD197^+^) was at least 90%. Isolated naive CD4^+^ T cells (7.5 × 10^4^ cells/well) were activated with Dynabeads Human T-Activator CD3/CD28 (2 μL/well; Thermo Fisher Scientific) and cultured with DCM derived from untreated (Ctl DCM) hDCs, EBV dUTPase–stimulated (EBV DCM) hDCs, or HHV-6A dUTPase–stimulated hDCs (HHV6 DCM), either alone or in combination with recombinant human IL-12p70 (IL-12, 5 ng/mL) for 3 and 5 days in X-VIVO 15 medium supplemented with recombinant human IL-7 (4 ng/mL). Cells treated with recombinant human-mouse-rat activin A (100 ng/mL) plus IL-12 were used as positive control. After 3 days, cells were collected for further analysis of T_FH_ differentiation by FACS as described below. IL-21 and IFN-γ levels in supernatants collected after 3 days of in vitro culture and following restimulation or not of differentiated cells with PMA (25 ng/mL, MilliporeSigma) plus ionomycin (1 μg/mL, MilliporeSigma) for 5 hours were determined by ELISA. For experiments quantifying the concentration of CXCL13 in culture supernatants, cells (7.5 × 10^4^ cells/well) were activated by plate-bound anti-human CD3 (5 μg/mL, clone OKT3) and ICOS-L (5 μg/mL, recombinant human B7-H2 Fc chimera) in the absence or presence of dUTPase DCM alone or in combination with IL-12. Supernatants from activated cells treated with activin A plus IL-12 were used as the positive control, and culture supernatants from anti-CD3/CD28 bead–activated as well as untreated cells were used as negative control. CXCL13 levels in supernatants collected after 5 days of in vitro culture were determined by ELISA.

For human activin blocking experiments, ME/CFS or control sera were preincubated with follistatin-315 (1 μg/mL, R&D Systems, Bio-Techne) or vehicle (DMSO) for 1 hour, added to freshly isolated naive CD4^+^ T cells, and incubated for 3 days at 37°C. On day 4, supernatants and cells were collected for analysis of IL-21 levels in the presence and absence of FST by ELISA and T_FH_ differentiation blockade by FACS. Activin A + IL-12 treatment in the absence of FST was used as a positive control, and bead-treated cells were used as the negative control.

### RayBiotech immune proteome array.

hDCs were seeded at a density of 2.5 × 10^5^ in 24-well plates and cultured in X-VIVO 15 serum-free medium supplemented with 500 U/mL GM-CSF and IL-4. The next day, hDCs were stimulated with EBV dUTPase or HHV-6A dUTPase proteins (10 μg/mL), or left untreated for 24 hours, as described previously (13, 14). Following treatments, culture supernatants were collected. The levels/concentration of 1000 immune mediators were measured using a Human L-1000 Antibody Array (RayBiotech), and the fluorescence was captured with an Axon GenePix laser scanner. Positive control spots on the array are standardized amounts of biotinylated IgGs printed directly onto the array. Negative control spots on the arrays contain Abs’ diluent buffer, and their signal intensities represent nonspecific binding of the Cy3-conjugated streptavidin (background signal). Normalized signal intensity data represent values in which the background signal of negative control spots has been subtracted out and normalized to the mean signal intensity of positive control spots. Following normalization, any ≥1.5-fold increase or ≤0.65-fold decrease in signal intensity for a single analyte between samples is considered a measurable and significant difference in expression, provided that both sets of signals are well above background (mean background + 2 SD, accuracy ≈ 95%). Data are expressed as the fold change signal intensity for each analyte in dUTPase-treated samples relative to the untreated control.

### Mice.

Female wild-type C57BL/6J mice (6 to 8 weeks of age) were purchased from The Jackson laboratory and housed for 7 days for acclimatization prior to use. Mice were housed in a Biosafety Level 2 barrier facility on a 12-hour light/12-hour dark cycle and given chow and water ad libitum. The facilities are maintained at 22°C–23°C and 30%–50% relative humidity. Mice (5–10 mice per treatment group) were injected daily with EBV dUTPase protein (10 μg/mL) or PBS control for 5–6 consecutive days. After 5–6 days, mice were sacrificed and spleens removed and processed for further analysis by FACS, qRT-PCR, ELISA, or immunohistochemistry (IHC).

### Flow cytometry.

Cells were run on a LSRII or LSR Fortessa (BD Biosciences) and analyzed with FlowJo software package (Tree Star). Cells were preincubated with FcR block (anti-CD16/CD32 mAb) prior to staining. For surface staining of human cells, the following fluorophore-conjugated mAbs were used: PerCP-eF710 CD4 (SK3), PE-Cy7 CD4 (RPA-T4), PE CD4 (RPA-T4), PE PD-1 (eBioJ105), and PE-Cy7 ICOS (ISA-3) from eBioscience, Thermo Fisher Scientific. BV421 CXCR5 (RF8B2) and APC CD4 (L200) were from BD Pharmingen. FITC CD45R (HI100) and APC CD197 (G043H7) were obtained from BioLegend. Fixable viability dyes FVD780 and FVD455UV were from eBioscience, Thermo Fisher Scientific. For intranuclear staining of human BCL6, cells were fixed with BD Cytofix Fixation Buffer, permeabilized with PhosFlow Permeabilization/Wash buffer I, and stained in permeabilization buffer with Alexa Fluor 647–BCL6 (K112-91).

For FACS analysis of mouse cells, single-cell suspensions of spleens were prepared in a gentleMACS OctoDissociator, using a mouse spleen dissociation kit (Miltenyi Biotec), followed by RBC depletion using TER-119 Ab (catalog 116204, BioLegend) (5 μg/spleen) and EasySep Mouse Streptavidin RapidSpheres Nanobeads (STEMCELL Technologies). For the identification of mouse B cell subsets, pan-B cells were isolated by magnetic separation using the EasySep Mouse Pan-B Cell Isolation by negative selection kit (STEMCELL Technologies), preincubated with mouse SeroBlock (Bio-Rad), and surface stained with fluorophore-conjugated mAbs specific for CD19 (6D5), CD23 (B3B4), IgD (11-26c.2a), and IgM (RMM-1) from BioLegend as well as CD21/CD35 (7G6) and CD9 (KMC8) from BD Pharmingen. For the identification of mouse iNKT_FH_ cells, spleen single-cell suspensions were depleted of CD19^+^ and CD8^+^ cells by magnetic separation using MojoSort CD19 and CD8a nanobeads followed by surface staining with the following fluorophore-conjugated mAbs: CD3e (145-2C11), B220 (RA3-6B2), CD11c (N418), CD11b (M1/70), and CD4 (RM4-5), all from BioLegend. PD-1 (J43) and Fas (Jo2) were from BD Pharmingen. FITC-conjugated PNA was from Vector Laboratories.

A 3-step staining of mouse CXCR5 was performed using a modification of a previously described method (51). Briefly, CD19- and CD8-depleted splenocytes were incubated with purified rat anti-mouse CXCR5 (2G8, BD Biosciences) for 45 minutes at RT followed by a 30-minute incubation with biotin-SP AffiniPure F(ab′)_2_ fragment mouse anti-rat IgG (H+L) (catalog 212-066-168, Jackson ImmunoResearch Laboratories) and then with BV421-labeled streptavidin (BioLegend) at 4°C in PBS + 0.5% BSA + 2% FBS + 2% normal mouse serum.

For intracellular staining of IL-21, spleen single-cell suspensions were prepared as described above and resuspended in 100 μL FACS buffer (PBS buffer with 2% BSA and 0.10% sodium azide) containing BD GolgiStop (1:800 dilution). Cells were fixed with BD Fixation/Permeabilization buffer and stained in permeabilization buffer with anti–IL-21 Ab (clone mhalx21, eBioscience, Thermo Fisher Scientific). For analysis of BCL6, cells were fixed and permeabilized with BD Pharmingen Transcription Buffer Set and stained with Abs against BCL6 (clone 7D1, BD Biosciences). Fluorescence minus one controls and isotype control Abs were used to set the gates.

### QRT-PCR analysis.

RNA was extracted using TRIzol reagent (Ambion) followed by RNeasy Mini Kit (QIAGEN) and on-column DNase treatment (QIAGEN). cDNA was synthesized using the SuperScript IV First-Strand Synthesis Kit (Invitrogen, Thermo Fisher Scientific), and qRT-PCR was performed on a QuantStudio 6 Flex instrument (Thermo Fisher Scientific) using the following TaqMan mouse gene-specific fluorogenic assays (Applied Biosystems, Thermo Fisher Scientific): *Cxcr5* (Mm00432086_m1), *Cxcl13* (Mm04214185_s1), *Cxcr4* (Mm01996749_s1), *Aicda* (Mm01184115_m1), *Fas* (Mm00433237_m1), *Pdcd1* (Mm01285676_m1), *Cd40* (Mm00441895_m1), *Cd40l* (Mm00441911_m1), *Icosl* (Mm00497237_m1), *Il21* (Mm00517640_m1), *Batf* (Mm00479410_m1), *Cd80* (Mm01344159_m1), *Il27* (Mm00461162_m1), *Cdkn1a* (Mm00432448_m1), *Tnfrsf13b* (Mm03047441_m1), *Tnfrsf14* (Mm00619237_m1), *Egr1* (Mm00656724_m1), *Flt3* (Mm00439016_m1), *Spib* (Mm03048233_m1), *Mcl1* (Mm01257351_g1), *Btla* (Mm00616981_m1), *Tnfrsf4* (Mm00442037_g1), *Pax5* (Mm00435501_m1), *Cd1d2* (Mm00776138_gH), *Xbp1* (Mm00457357_m1), *Tnfsf13b* (Mm00446347_m1), *Il15* (Mm00434210_m1), *Ebf1* (Mm00432954_m1), *Cd38* (Mm00483143_m1), *B2m* (Mm00437762_m1), and *Hsp90ab1* (Mm00833431_g1). All reactions were carried out in a final volume of 20 μL containing 10 μL of TaqMan gene expression master mix. Samples were normalized to internal standards (*B2m* and *Hsp90ab1*) and expressed as mRNA expression levels relative to the vehicle control. All reactions were performed in triplicate. The fold change/fold regulation of the expression for each target gene was calculated with the threshold cycle (Ct) values as follows: fold change (2^-ΔΔCt^) is the normalized gene expression (2^-ΔCt^) in the test sample divided by the normalized gene expression (2^-ΔCt^) in the control sample. Fold change values greater than 1 indicate an upregulation, and the fold regulation is equal to the fold change. Fold change values less than 1 indicate a downregulation, and the fold regulation is the negative inverse of the fold change.

### Mouse IHC.

IHC was performed at The Ohio State University Comparative Pathology and Mouse Phenotyping Shared Resource and the The Ohio State University Wexner Medical Center Pathology Core facilities. Immunoperoxidase staining of spleen sections was performed on formalin-fixed, paraffin-embedded sections (4 μm) from EBV dUTPase– or PBS-injected mice. Briefly, tissue sections were deparaffinized, rehydrated, and subjected to antigen retrieval by heat-induced epitope retrieval (Dako; pH 6.0) for 25 minutes at 96°C and cooled for 15 minutes. Slides were stained with the primary Ab rat anti–mouse CD4 (5 μg/mL), for 1 hour at RT using the Intellipath Autostainer Immunostaining instrument. Rat isotype control was used as a negative control. The stained spleen sections were washed and incubated with goat anti-rat HRP-conjugated secondary Ab (catalog 205720, Abcam). Bound Abs were visualized by incubation with 3,3′-diaminobenzidine chromogen (liquid DAB+) for 5 minutes at RT. Spleen slides were counterstained with Richard-Allan Scientific (Thermo Fisher Scientific) hematoxylin, dehydrated, cleared, and mounted. All slides were scanned using the Evos FL Auto 2 digital imaging system (Software Version 2.0.883.0), or images were obtained with a Leica DM5000 B microscope equipped with Leica Application Suite software (Version 4.2.0, Leica Microsystems).

### H&E staining.

Spleen tissue sections (4 μm) were stained with hematoxylin (Richard-Allan Scientific, Thermo Fisher Scientific) and eosin (Eosin-Y, Epredia Richard-Allan Scientific, Thermo Fisher Scientific) using a Leica Autostainer XL (Leica Biosystems) for evaluation of general tissue architecture.

### Statistics.

All statistical analyses were performed with GraphPad Prism 9 or Ingenuity Pathway Analysis (IPA) software. All data represent mean ± SEM. For all analyses, comparisons between treatment and/or disease groups versus controls are shown unless otherwise indicated in the figure legends. For statistical analysis comparing 2 groups, a 2-tailed Mann-Whitney *U* test was used. For multiple-comparison analyses, a 1-way ANOVA Kruskal-Wallis multiple-comparison test with Dunn’s correction was used. Values of *P* < 0.05 were considered significant. For determining statistical significance of pathway associations in IPA, right-tailed Fisher’s exact test followed by Benjamini-Hochberg method correction was used. –Log_10_-transformed *P* > 1.3 was considered statistically significant. The number of experiments, animals per group, and the statistical test used are indicated in the figure legends or in the appropriate text.

### Study approval.

All human serum samples used in this study were deidentified and approved by the IRB of The Ohio State University (OSU). All participants provided written informed consent, and the protocol was approved by the IRB committee for human subjects of Nova Southeastern University, Fort Lauderdale, Florida, USA. OSU Animal Care and Use Committee approved the protocol, in keeping with the regulations of the American Physiological Society.

## Author contributions

MEA devised the study and obtained funding. MEA, BSC, KA, IMP, and WPL designed and conducted the experiments and analyzed data. MEA wrote the paper; BSC, KA, IMP, and WPL edited the paper.

## Figures and Tables

**Table 3:**
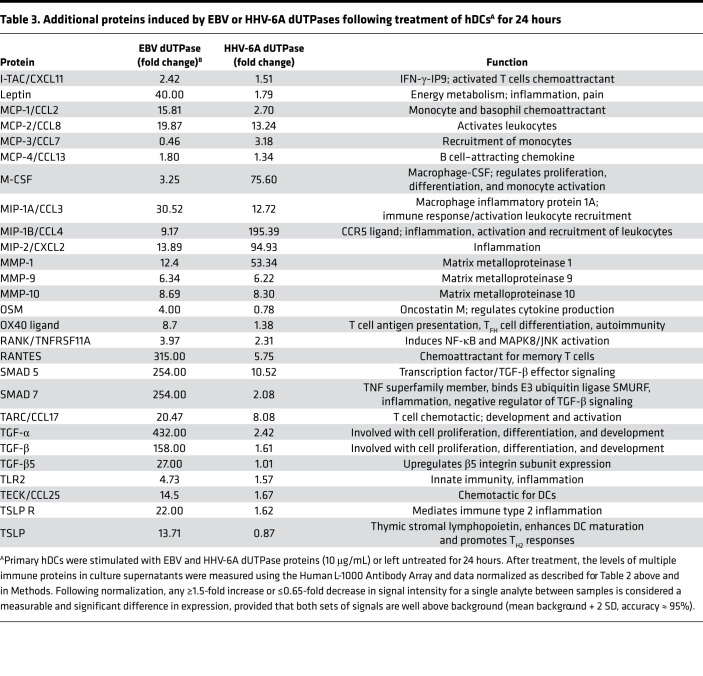
Additional proteins induced by EBV or HHV-6A dUTPases following treatment of hDCs^A^ for 24 hours

**Table 2:**
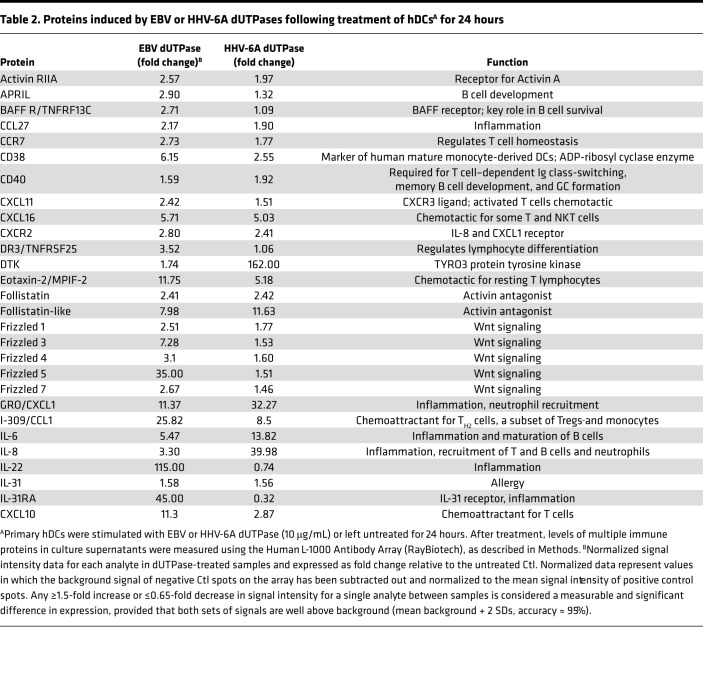
Proteins induced by EBV or HHV-6A dUTPases following treatment of hDCs^A^ for 24 hours

**Table 1:**
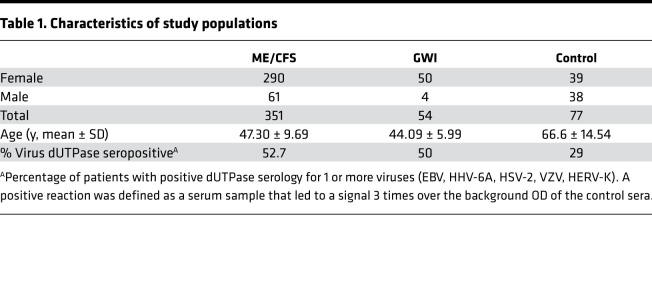
Characteristics of study populations

## Version 1 04/28/2022

In-Press Preview

## Version 2 06/08/2022

Electronic publication
